# Inhibition of microfold cells ameliorates early pathological phenotypes by modulating microglial functions in Alzheimer’s disease mouse model

**DOI:** 10.1186/s12974-023-02966-9

**Published:** 2023-11-27

**Authors:** Namkwon Kim, In Gyoung Ju, Seung Ho Jeon, Yeongae Lee, Min-Ji Jung, Min Sung Gee, Jae Seok Cho, Kyung-Soo Inn, Lee Ann Garrett-Sinha, Myung Sook Oh, Jong Kil Lee

**Affiliations:** 1 Department of Life and Nanopharmaceutical Sciences, Graduate School, Kyung Hee University, Seoul, Republic of Korea; 2 Department of Pharmacy, College of Pharmacy, Kyung Hee University, Seoul, Republic of Korea; 3 Kyung Hee East-West Pharmaceutical Research Institute, Kyung Hee University, Seoul, Republic of Korea; 4 Department of Oriental Pharmaceutical Science, College of Pharmacy, Kyung Hee University, Seoul, Republic of Korea; 5 Department of Pharmaceutical Science, College of Pharmacy, Kyung Hee University, Seoul, Republic of Korea; 6 Department of Biochemistry, State University of New York at Buffalo, Buffalo, NY USA

**Keywords:** Alzheimer’s disease, Microbiota-Gut-Brain axis, Microfold cells, Microglia

## Abstract

**Background:**

The gut microbiota has recently attracted attention as a pathogenic factor in Alzheimer’s disease (AD). Microfold (M) cells, which play a crucial role in the gut immune response against external antigens, are also exploited for the entry of pathogenic bacteria and proteins into the body. However, whether changes in M cells can affect the gut environments and consequently change brain pathologies in AD remains unknown.

**Methods:**

Five familial AD (5xFAD) and 5xFAD-derived fecal microbiota transplanted (5xFAD-FMT) naïve mice were used to investigate the changes of M cells in the AD environment. Next, to establish the effect of M cell depletion on AD environments, 5xFAD mice and *Spib* knockout mice were bred, and behavioral and histological analyses were performed when M cell-depleted 5xFAD mice were six or nine months of age.

**Results:**

In this study, we found that M cell numbers were increased in the colons of 5xFAD and 5xFAD-FMT mice compared to those of wild-type (WT) and WT-FMT mice. Moreover, the level of total bacteria infiltrating the colons increased in the AD-mimicked mice. The levels of M cell-related genes and that of infiltrating bacteria showed a significant correlation. The genetic inhibition of M cells (*Spib* knockout) in 5xFAD mice changed the composition of the gut microbiota, along with decreasing proinflammatory cytokine levels in the colons. M cell depletion ameliorated AD symptoms including amyloid-β accumulation, microglial dysfunction, neuroinflammation, and memory impairment. Similarly, 5xFAD-FMT did not induce AD-like pathologies, such as memory impairment and excessive neuroinflammation in *Spib*^−/−^ mice.

**Conclusion:**

Therefore, our findings provide evidence that the inhibiting M cells can prevent AD progression, with therapeutic implications.

**Supplementary Information:**

The online version contains supplementary material available at 10.1186/s12974-023-02966-9.

## Introduction

Alzheimer’s disease (AD) is an irreversible and progressive neurological disease that presents with symptoms of memory and cognitive impairment due to brain shrinkage, or atrophy. Typical pathological hallmarks include senile plaques of aggregated extracellular amyloid-β (Aβ) and neurofibrillary tangles of hyperphosphorylated tau protein [1]. Additionally, excessive neuroinflammation is considered a major disease-driving factor [2]. Infiltrating peripheral immune cells, including T cells, are also recognized as factors that can influence AD pathology [3]. Despite intensive efforts to elucidate the etiology of this disease, the key molecular mechanisms underlying the development of AD remain largely unknown.

In the past decade, interest has been increased on the microbiota-gut-brain (MGB) axis, a theory that changes of gut microenvironments can impinge on host brain activity and behavioral function. For instance, patients with irritable bowel syndrome (IBS) exhibit depression-like symptoms [4]. Transplantation of fecal microbiota from patients with IBS deteriorates gastrointestinal motility and intestinal barrier function in mice, resulting in anxiety-like behavior [5]. Dextran sulfate sodium (DSS)-induced colonic inflammation decreases hippocampal neurogenesis, along with increased neuroinflammation [6]. Furthermore, our previous study showed that fecal microbiota transplantation (FMT) from five familial AD (5xFAD) mice to naïve mice (5xFAD-FMT) increases colonic expression of proinflammatory cytokines, resulting in cognitive dysfunction and decreased neurogenesis [7]. Treatment with *Escherichia coli* or lipopolysaccharide (LPS) isolated from *E. coli* also causes neuroinflammation and reduces neurogenesis, leading to a memory decline in the mice [8, 9]. Alteration of gut microbiota induced by antibiotics (ABX) treatment has been shown to ameliorate impaired brain function in AD mouse models [10]. Although the MGB axis is considered a key marker influencing the pathogenesis of neurodegenerative diseases, its molecular mechanism link is yet elusive.

Microfold (M) cells, which comprise a specialized subset of intestinal epithelial cells, are characterized by irregular microvilli and pocket-like basolateral invaginations. Most M cells usually exist in the follicle-associated epithelia, such as Peyer’s patches (PPs). They play a pivotal role in mucosal immune surveillance by transporting harmful external proteins and microbiota to underlying immune cells [11]. Meanwhile, M cells are used as the entry sites by foreign antigens, including bacteria and proteins, and their increased density makes them susceptible to infection [12–14]. In this aspect, M cell counts can be increased by external stimuli such as the cholera toxins, bacteria-derived protein, and proinflammatory cytokines, which are distinctively identified as “inducible M cells”[15, 16].

Given the aforementioned concepts and findings, we hypothesized that if the number of M cells differed between normal and AD microenvironments, then its regulation may influence the pathogenesis of AD. To explore the role of the M cells in AD microenvironments, we investigated changes for brain and gut environments using AD-mimicked mice (5xFAD and 5xFAD-FMT) and M cell-depleted AD-mimicked mice (5xFAD/*Spib*^−/−^ and 5xFAD-FMT/*Spib*^−/−^).

## Materials and methods

### Mice

The 5xFAD (B6SJL) and control (wild-type, WT; B6SJL) mice were purchased from the Jackson Laboratory (Bar Harbor, ME, USA). We obtained 8-week-old male and female *Spib* (C57BL/6) heterozygous mice from Prof. Lee Ann Garrett-Sinha (the State University of New York at Buffalo). *Spib*^±^ mice were bred with 5xFAD mice to generate WT/*Spib*^±^ and 5xFAD/*Spib*^±^ mice. Data obtained from the WT, WT/*Spib*^−/−^, 5xFAD, and 5xFAD/*Spib*^−/−^ mice were analyzed when they were 6–7 months old. The FMT mouse model was divided into 4 groups (Fig. 6A): WT-FMT (C57BL/6 mice transplanted with fecal microbiota from WT mice), WT-FMT/*Spib*^−/−^ (M cell-depleted mice transplanted with fecal microbiota from WT mice), 5xFAD-FMT (C57BL/6 mice transplanted with fecal microbiota from 5xFAD mice), 5xFAD-FMT/*Spib*^−/−^ (M cell-depleted mice transplanted with fecal microbiota from 5xFAD mice). The mice were housed in plastic cages under the conditions of constant temperature (23 ± 1 °C), humidity (50 ± 10%), 12 h light/dark cycle, and access to diet and water available ad libitum. The diet information is shown in Additional file 1: Table S1. The block randomization method was used to allocate the mice to experimental groups. To eliminate the bias, we were blinded to the experimental progress, including behavioral test and quantitative analysis. All animal studies were performed in accordance with the “Guide for the Care and Use of Laboratory Animals, 8^th^ edition” of the National Institutes of Health (2011) and approved by the “Animal Care and Use Guidelines” of Kyung Hee University (approval number: KHUASP(SE)-18–145 and KHUASP(SE)-20–433).

### Y-maze test

The test apparatus consisted of three arms (3 × 40 × 12 cm) positioned at an angle of 120° away from each other. Mice were placed in the middle of the Y-maze and allowed to explore the three arms for 8 min. All mouse behaviors were recorded using a camera, and the order in which they entered the three arms was manually checked. The numbers and sequences of the arm entries were measured. The percentage alternation, which is a measure of spatial working memory, was calculated as follows: Spontaneous alternations (%) = [Total number of alternations / (Total number of arm entries – 2) × 100. Alternation behavior was defined as consecutive entries into all three arms (i.e., ABC, CAB, or BCA, but not BAB).

### Morris water maze (MWM) test

We performed the Morris water maze (MWM) test to evaluate the spatial memory performance. The water maze was a white tank (1.0 m diameter, 30 cm height) filled with water to a depth of 20 cm (22–24 °C). White, opaque, and nontoxic paint was added to the water to hinder visibility. A submerged Plexiglas platform (10 cm diameter, 6–8 mm below the surface of the water) was placed at a fixed position throughout the training session. The position of the platform varied for the mice but was counterbalanced across the experimental groups. All mice were habituated to the maze 1 day before training and were subjected to three trials per day. In Fig. 2, the training session of the experimental group consisted of three trials per day for 10 consecutive days (a total of 30 trials), in Fig. 6, the training session of the experimental group consisted of two trials per day for 6 consecutive days (a total of 12 trials). In each the trials, the mice were randomly placed at different starting positions that were equally spaced around the perimeter of the pool. Mice were allowed to stand for 60 s to find the submerged platform. If the mice did not mount the platform within 60 s, they were guided to the platform. The time taken to mount the platform was recorded as the latency for each trial. All the mice were allowed to remain on the platform for 10 s before being returned to their home cages. A single-probe trial, in which the platform was removed, was performed after the hidden platform task had been completed (mice of Fig. 2; days 11, mice of Fig. 6; days 7). Each mouse was placed in one quadrant of the pool and allowed to swim for 60 s. All trials were recorded using a charge-coupled device camera connected to a video monitor and computer. The escape time, crossing the platform number, and spent time in the target quadrant of the muse were manually checked, and swimming speed, total distance, and swimming paths were analyzed using a ToxTrac software.

### Brain histological analysis

After the behavioral test, the mice were anesthetized and sacrificed. The mice were immediately cardiac-perfused with 1X phosphate-buffered saline (PBS) and 4% paraformaldehyde (PFA). After perfusion, brains of mice were removed and postfixed with 4% PFA overnight at 4 °C, and then incubated in 30% sucrose at 4 °C until equilibrated. Next, sequential 30-μm coronal sections were cut on a freezing microtome (Leica, Wetzlar, Germany) and stored at −20 °C.

Free-floating brain sections were rinsed with 1X PBS, and then incubated with 1X PBS containing 1% bovine serum albumin (BSA), 3% normal goat serum, and 0.4% Triton X-100 for 1 h at room temperature (RT). The sections were incubated with the primary antibody overnight at 4 °C in the same buffer solution. The following primary antibodies were used: anti-6E10 (1:500, BioLegend, 803,001), anti-4G8 (1:500, BioLegend, 800701), anti-ionized calcium-binding adapter molecule-1 (Iba-1; 1:500, Wako, 019-19741), anti-glial fibrillary acidic protein (GFAP; 1:1000, Dako, Z0334), anti-CD68 (1:500, Abcam, ab53444). After rinsing 1X PBS, we visualize with secondary antibodies diluted in blocking buffer for 1 h at RT. The following secondary antibodies were used: anti-mouse Alexa Fluor 594 (1:1000, Invitrogen, A11005), anti-mouse Alexa Fluor 405 (1:1000, Invitrogen, A31553), anti-rabbit Alexa Fluor 594 (1:1000, Invitrogen, A11012), anti-rabbit Alexa Fluor 488 (1:1000, Invitrogen, A11008), and anti-rat Alexa Fluor 594 (1:1000, Invitrogen, A11007).

For Thioflavin S (ThS) staining, free-floating brain sections were incubated at a 0.5% ThS (in 50% EtOH) for 5 min, and then washed 50% EtOH, 70% EtOH, and 100% EtOH for 5 min, and mounted with mounting medium.

The sections were photographed using a K1-Fluo confocal microscope (Nanoscope Systems, Daejeon, Republic of Korea) or with an Olympus BX51 Fluorescence Microscope (Olympus, Tokyo, Japan). The positive area (%) of fluorescence signal was quantified using the Image J software (Bethesda, MD, USA).

### Glycoprotein 2 (GP2) immunostaining

Intestinal tissues were 4% PFA fixed immediately after dissection and paraffin was embedded after 24 h. Using a microtome (Accu-Cut® SRM™ 200; Sakura, Torrance, CA, USA), sequential 8-µm-thick tissue were sectioned and mounted on gelatin-coated glass slides. To stain mature M cells in the colon using glycoprotein (GP2) antibody (MBL International Corporation, Woburn, MA, USA; D278-3), deparaffinized sections were incubated with blocking buffer (1% BSA, 3% normal goat serum, and 0.4% Triton X-100 in 1X PBS) for 1 h at RT. The sections were incubated with the GP2 antibody overnight at 4 °C in the blocking buffer. After rinsing 1X PBS, the sections were visualized with a secondary antibody (anti-rat Alexa Fluor 594) diluted in blocking buffer for 1 h at RT. After five times rinses with 1X PBS, the sections were stained with DAPI for 20 min and mounted on gelatin-coated slide with a fluorescent mounting medium. The sections were analyzed using a K1-Fluo confocal microscope. The number of GP2-positive cells was quantified as an average by capturing 20 sites from four tissues of one mouse.

### Hematoxylin and eosin staining

Paneth cells of the colon were analyzed using hematoxylin (SH3777) and eosin (EM500G) staining (cancer diagnostics) were performed according to the manufacturer’s protocols.

### Alcian blue staining

Goblet cells of the colon were analyzed using alcian blue staining kits (Abcam, ab150662) according to the manufacturer’s protocols.

### Aβ enzyme-linked immunosorbent assay (ELISA)

For measurement of Aβ_1-40_ and Aβ_1-42_ levels of the brain, we used commercially available ELISA kits (Thermo Fisher Scientific, Waltham, MA, USA; KHB3481, KHB3441). Hemispheres of mice were homogenized in a 5 M guanidine buffer. ELISA was then performed for Aβ_1-40_ and Aβ_1-42_ according to the manufacturer’s instructions.

### Western blot analysis

The hippocampus and cortex were weighed and lysed in 8 × volumes of RIPA lysis buffer (Thermo Fisher, 89901) plus protease and phosphatase inhibitors (Thermo Fisher, 78444). Equal amounts of protein samples (20 or 30 μg) were dissolved in the sample buffer. The heat-treated samples were subjected to sodium dodecyl sulfate–polyacrylamide gel electrophoresis and transferred electrophoretically to immunoblotting PVDF membranes. The membranes were then pretreated with the blocking solution (5% (w/v) dry skim milk or 5% BSA, 0.1% Tween 20 in Tris-buffered saline, TBS) for 1 h at RT and incubated with primary antibodies in the blocking solution overnight at 4 °C. The following primary antibodies were used: anti-6E10 (1:1000, BioLegend, 803001), anti-a disintegrin and metalloproteinase 10 (ADAM10; 1:1000, Santa Cruz, sc-48400), anti-presenilin-1 (PS1; 1:2000, Cell signaling, 5643 s), anti-insulin-degrading enzymes (IDE; 1:2000, Abcam, ab32216), anti-neprilysin (NEP; 1:500, R&D Systems, AF1126), anti-zonula occludens-1 (ZO-1; 1:1000, Invitrogen, 40-2200), anti-occludin (1:500, Invitrogen, 33–1500), anti-β-actin (1:10,000, Santa Cruz, sc-47778HRP). Next, the membranes were washed with a washing solution (0.1% Tween 20 in TBS) 5 times for 5 min each and incubated with horseradish peroxidase (HRP)–conjugated secondary antibodies against mouse, rabbit, or goat IgG in the blocking solution for 1 h at RT. The following secondary antibodies were used: anti-mouse HRP (1:5000, Santa Cruz, sc-516102), anti-rabbit (1:5000, Santa Cruz, sc-2357), anti-goat (1:2000, Santa Cruz, sc-2020). The membranes were then washed with the washing solution 5 times for 5 min each. The protein signals were developed with an enhanced chemiluminescence reagent (Bio-Rad Laboratories, Hercules, CA, USA) and visualized using a FUSION Solo 6S EDGE (Vilber Lourmat St´e, Coll´egien, France). We then detected β-actin in the same blot as an internal control for normalizing protein loading. The intensity of the bands was quantified using the Image J software.

### RNA isolation and quantitative real-time polymerase chain reaction (qRT-PCR)

Total RNA was extracted and purified from the hippocampus, cortex, colon, or PPs using the Hybrid-R™ (GeneAll, Seoul, Republic of Korea). Before measuring total bacteria level of the colons, they were incubated with gentamicin (100 μg/mL; Gibco, 15710064) at 4 °C for 30 min to remove external bacteria. Its concentration was measured using a NanoDrop ND-2000 spectrophotometer (Thermo Fisher). Next, RNA samples (3 μg) were converted to complementary DNA using TOPscript™ RT DryMIX (Enzynomics, Daejeon, Republic of Korea). The cDNA was subjected to qRT-PCR using TOPreal™ qPCR 2X PreMIX (SYBR Green; Enzynomics) and the CFX Connect Real-Time PCR System (Bio-Rad Laboratories). All genes were quantified relative to a reference gene, glyceraldehyde 3-phosphate dehydrogenase (GAPDH), and relative mRNA levels were calculated using the 2^−ΔΔct^ method. Primers synthesized at Cosmo Genetech (Seoul, Republic of Korea). The primer sequences are shown in Additional file 1: Table S2.

### Assessment of microglial functions in vivo

For the in vivo Aβ phagocytosis assay, mice were intraperitoneally injected 3 h before being killed with 10 mg/kg methoxy-X04 (MeX04) working solution (2 mg/mL; Tocris, 4920) in 50% DMSO/50% NaCl (0.9%, pH 12) 3 h before being killed. The subsequent step followed the flow cytometry protocol.

### Flow cytometry

Immune cells in the brain were analyzed using flow cytometry. Cells from WT, WT/*Spib*^−/−^, 5xFAD, and 5xFAD/*Spib*^−/−^ mouse brains were prepared as previously described [17], with minor modifications. The brains of the WT, WT/*Spib*^−/−^, 5xFAD, and 5xFAD/*Spib*^−/−^ mice were dissected and immediately transferred to ice-cold 1X Hank’s balanced salt solution (HBSS) (Welgene, Gyeongsan, Republic of Korea; LS203-06). After gently separating the brains into small pieces, they were digested in an HBSS solution containing HEPES (2 mM; Hyclone Laboratories, Logan, UT, USA; SH30237.01), collagenase D (0.5 mg/mL; Sigma Aldrich, St. Louis, MO, USA; 11088866001), and DNase I (0.5 mg/mL; Sigma Aldrich, DN25) at 37 °C for 1 h under gentle rocking. Digestion was stopped by adding fetal bovine serum (Gibco, Waltham, MA, USA; 10082139) on ice. Using a sterile plunger with a 5 cc syringe, the dissociated brains were gently passed through a cell strainer (40 μm pore-size). The supernatants were centrifuged at 400 × *g* for 5 min at 4 °C. The pellet was resuspended in 25% Percoll (Sigma Aldrich, P4937) for myelin removal. After centrifugation step at 400 × *g* for 20 min at 4 °C, the myelin-containing supernatant was discarded. The cell pellets were resuspended in red blood cell lysis buffer (Sigma Aldrich, 11814389001) and incubated at RT for 5 min to lyse the erythrocytes. The cells were washed with a FACS buffer (containing 1% BSA and 1 mM EDTA in sterile 1X PBS) at 400 × g for 5 min at 4 °C.

The cells isolated from brain were stained with the following antibodies for T cells, B cells, or dendritic cells: anti-CD45 PE-Cy5.5 (1:100, BD Bioscience, 561870), anti-CD45 APC-Cy7 (1:100, BioLegend, 103116), anti-CD4 FITC (1:100, BioLegend, 100406), anti-CD8 APC-Cy7 (1:100, BD Bioscience, 557654), anti-CD11b APC (1:100, BD Bioscience, 553312), anti-CD11b APC-Cy7 (1:100, BD Bioscience, 557657), anti-CD11c PE-Cy7 (1:100, BD Bioscience, 558079), anti-B220 BV421 (1:100, BioLegend, 103240). Permeabilization, fixation, and staining of intracellular marker with anti-IL-4 PE (1:50, BioLegend, 504104), anti-IFN-γ APC (1:50, BioLegend, 505810), anti-IL-17 PerCP-Cy5.5 (1:50, BioLegend, 506920), anti-CD68 Alexa488 (1:50, BioLegend, 137012) were performed with Inside Stain kit (Miltenyi Biotec, 130-090-477) according to the manufacturer’s instructions. After an incubation period of 1 h at 4 °C, fluorescence data were acquired on a Cyto FLEX (Beckman Coulter, Brea, CA, USA) using CytExpert software (Beckman Coulter).

### Bacterial sequencing

Bacterial sequencing analyses were performed by Macrogen Inc. (Seoul, Republic of Korea). Fecal DNA of five male 6-months-old mice was extracted using the Dneasy PowerSoil Kit (Qiagen, 12888–100) according to the manufacturer’s protocol. Each sample to be sequenced was prepared according to the Illumina 16S metagenomic sequencing library protocols. The quantity and quality of the DNA were measured by PicoGreen using VICTOR Nivo (PerkinElmer, Waltham, MA, USA). The 16S rRNA genes were amplified using 16S V3-V4 primers (forward 5′-CCTACGGGNGGCWGCAG-3′ and reverse 5′-GACTACHVGGGTATCTAATCC-3′). Input gDNA was amplified using 16S V3-V4 primers, and a subsequent limited-cycle amplification step was performed to add multiplexing indices and Illumina sequencing adapters. The final products were normalized and pooled using PicoGreen, and the sizes of the libraries were verified using the TapeStation DNA screentape D1000 (Agilent Technologies, Santa Clara, CA, USA). Sequencing was performed using the MiSeq platform (Illumina, San Diego, CA, USA).

### Fecal microbiota transplantation (FMT)

FMT was performed as previously described procedures [7]. Fresh fecal pellets were collected from 9-month-old WT (*n* = 5) and 5xFAD (*n* = 5) male mice. The feces (1.5 g) of five mice collected from each group were then mixed, steeped, and shaken in sterile 1X PBS (pH 7.4, 10 mL), and then filtered through a 100 μm pore mesh. The final bacterial suspension was stored at −80 °C. Before administering the bacterial suspension, to reduce the commensal microbiota of the colon, *Spib*^+/+^ and *Spib*^−/−^ mice were provided with sterile water (ad libitum) containing 2000 U/mL penicillin and 2 mg/mL streptomycin for 3 days to reduce the commensal microbiota of the colon, and then washed out for 3 days. Next, 200 μL of fecal microbiota was administered for five consecutive days via oral gavage.

### Statistical analysis

The results are presented as mean ± standard error of the mean or standard deviation. The results were analyzed using a two-way analysis of variance (ANOVA), with the least significant difference test among the four groups. The differences between the two groups were analyzed with Students’ *t*-test. Results with *P* < 0.05 were considered statistically significant. All statistical parameters were calculated using the GraphPad Prism ver.8.0 (GraphPad Software, San Diego, CA, USA).

## Results

### M cell counts in the colon are increased in the AD microenvironments

**Fig. 1:**
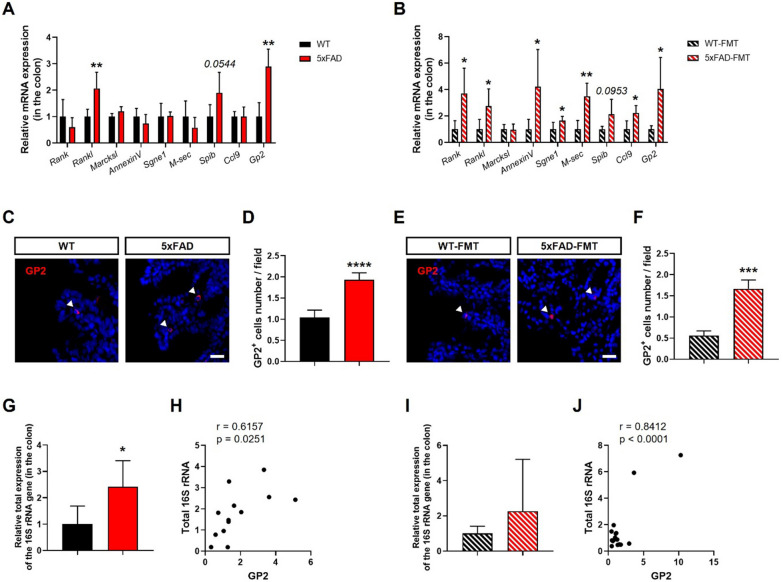
Microfold cell counts were increased in the colons of 5xFAD and 5xFAD-FMT mice. **A**, **B** Expression of M cells-related genes in the colons of 5xFAD **A** and 5xFAD-FMT **B** mice (*n* = 4–5 per group). **C** and **E** Representative images of GP2^+^ cells in the colons of 5xFAD **C** and 5xFAD-FMT **E** mice; scale bar, 20 μm. The quantification of GP2^+^ cells in the colons of 5xFAD **D** and 5xFAD-FMT **F** mice (*n* = 3–5 per group). **G** and **I** The level of total bacteria was measured in the colons of 5xFAD **G** and 5xFAD-FMT **I** using qRT-PCR (*n* = 5–9 per group). **H**, **J** Correlation analysis of total bacteria and *Gp2* was analyzed in the colons of WT and 5xFAD mice **H** and WT-FMT and 5xFAD-FMT mice **J**. Bars represent as mean ± standard deviation. Statistical analysis included the Student’s *t*-test. *P < 0.05, **P < 0.01, ***P < 0.001, ****P < 0.0001 *vs. *WT or WT-FMT mice

The number of M cells is known to increase during intestinal inflammation. Patients with ulcerative colitis harbor an increased number of M-like cells in the colon [18]. DSS-induced colonic inflammation was also found to increase the number of inducible M cells [16]. We previously reported that the administration of 5xFAD-derived gut microbiota induces inflammation in the colons of naïve mice [7]. Therefore, we first investigated whether the number of M cells in the colon changed in the AD environments. In the colons of 5xFAD and 5xFAD-FMT mice, expression of M cell-related genes was increased compared to those in WT and WT-FMT mice, respectively (Fig. 1A, B). In particular, the mRNA levels of the receptor activator of nuclear factor-κB ligand (*Rankl*; an M cell differentiation factor), *Spib* (an M cell transcription factor), and *Gp2* (a mature M cell marker) were nearly doubled in the AD microenvironment compared to those in the health microenvironment. To confirm whether mature M cell counts were increased in the colons of AD-mimicked mice, we performed an immunofluorescence assay using a GP2 antibody. We observed that the number of GP2-positive cells was significantly increased in the colons of 5xFAD and 5xFAD-FMT mice (Fig. 1C–F), indicating that colonic M cell counts increased in the AD microenvironments.

Since bacteria use M cells to invade the tissues, we expected that the number of infiltrating bacteria in the colons of AD-mimicked mice was higher than that of controls. We found that the level of total bacteria was increased in the colons of AD-mimicked mice compared to that of WT or WT-FMT mice (Fig. 1G, I). The level of *Gp2* was correlated significantly with the level of total bacteria in the colons of all groups (Fig. 1H and J). These results indicate that there was an increase in bacteria infiltrating intestine through M cells in the AD microenvironments.

We further investigated the number of PPs and the expression of M cell-related genes within them, since most typical M cells exist in PPs, and their numbers can decrease with aging. No difference was noted in PPs between the 5xFAD and 5xFAD-FMT mice and the WT and WT-FMT mice (Additional file 1: Fig. S1A and C). In the PPs of 5xFAD-FMT mice, expression of M cell-related genes did not exhibit changes, but expression levels of *Rankl*, *Spib*, and *Gp2* were upregulated in the 5xFAD mice compared with those in the WT mice (Additional file 1: Fig. S1B and D).

The disruption of the tight junction barriers increases the invasion of harmful bacteria into the body. To observe the barrier integrity, we examined the protein levels of tight junction proteins (ZO-1 and occludin) in the colons of mice. There were no significant changes in the colonic tight junctions between the AD and normal microenvironments in our experimental design (Additional file 1: Fig. S2). Next, we investigated whether there were changes in other cells that played specific roles in the colons, such as goblet cells and Paneth cells, in the AD microenvironments. To characterize these cells in the colons, we conducted Alcian blue and hematoxylin and eosin staining. These cells did not show significant differences in the normal and AD microenvironment (Additional file 1: Fig. S3). Altogether, these analyses showed that there were no significant changes in the expression of the tight junction proteins and other cells, except M cells, in AD microenvironments.

### M cell inhibition reduces amyloid-β accumulation, resulting in improvement of memory function

To assess the relationship between increased M cell counts and AD pathology, we generated 5xFAD/*Spib*^−/−^ mice (5xFAD mice with a genetic deletion of the *Spib* gene). *Spib* is a transcription factor specifically expressed in M cells, and *Spib*^−/−^ mice have been used in M cell-related research [19]. We divided the offspring into four groups by performing genotyping: WT, WT/*Spib*^−/−^, 5xFAD, and 5xFAD/*Spib*^−/−^ mice (Additional file 1: Fig. S4A). We confirmed that the expression levels of *Spib* and *Gp2* were reduced in the colons of *Spib*^−/−^ mice (Additional file 1: Fig. S4B and C). There were no significant changes in the body weights of either female or male WT/*Spib*^−/−^ and 5xFAD/*Spib*^−/−^ mice (6 months old) compared to WT and 5xFAD mice (Additional file 1: Fig. S4D and F). Therefore, 5xFAD/*Spib*^−/−^ mice were considered suitable for evaluating the correlation between M cells and AD pathology.

**Fig. 2:**
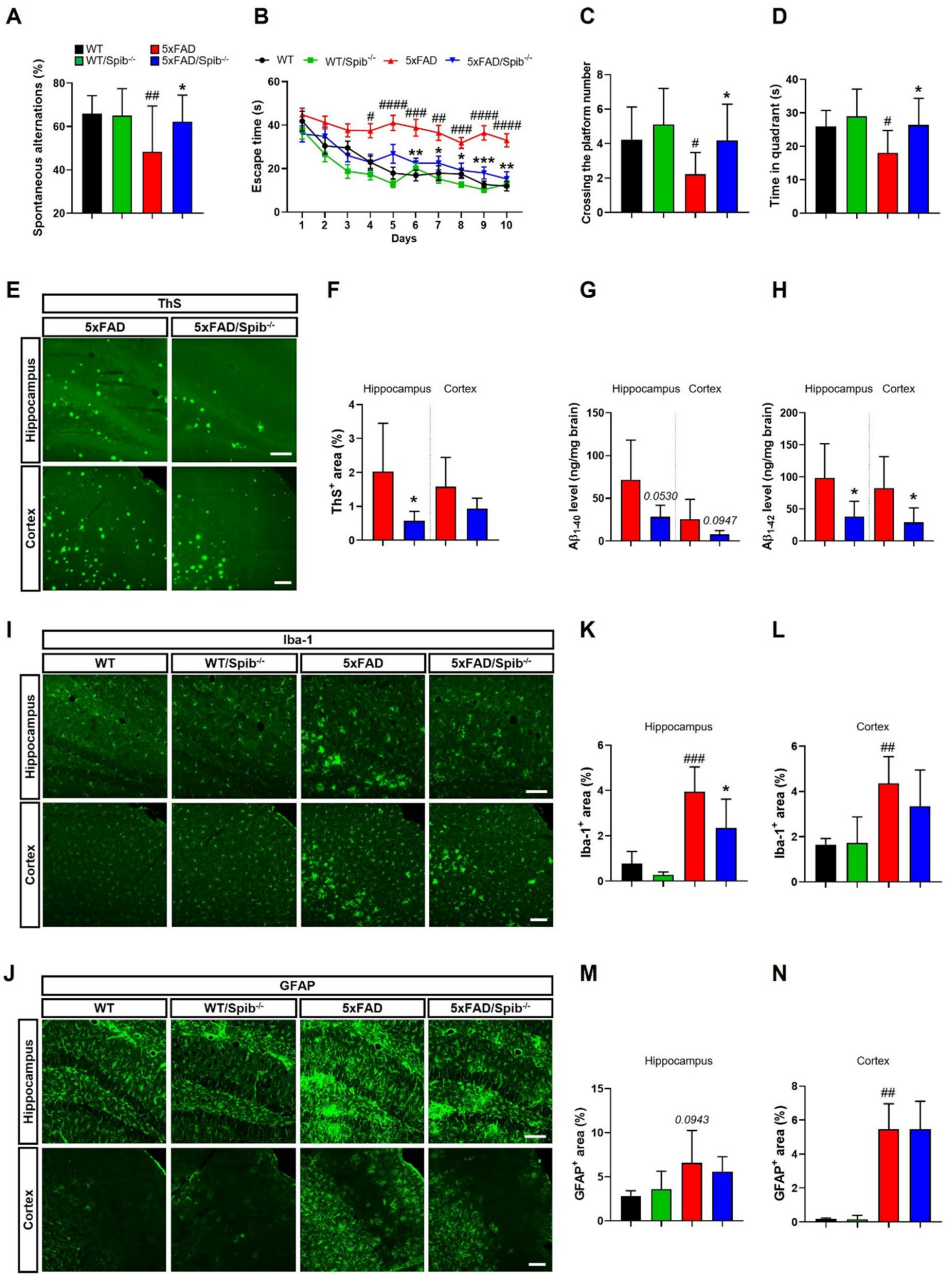
Depletion of microfold cells alleviated memory dysfunction and Aβ pathologies in 5xFAD mice. **A** Spontaneous alternations (%) of WT, WT/*Spib*^−/−^, 5xFAD, and 5xFAD/*Spib*^−/−^ mice (WT, *n* = 17; WT/*Spib*^−/−^, *n* = 17; 5xFAD, *n* = 18; 5xFAD/*Spib*^−/−^ mice, *n* = 16). **B** Escape latencies of WT, WT/*Spib*^−/−^, 5xFAD, and 5xFAD/*Spib*^−/−^ mice over 10 days. **C**, **D** In the probe trial on day 11, crossing the platform number **C** and spending time in the target quadrant **D** were recorded and analyzed. Statistical analysis included the two-way ANOVA and Tukey’s post hoc test. Brain sections from 6-month-old mice were stained with thioflavin S (ThS). **E** Representative images of ThS staining of the hippocampus and cortex; scale bar, 100 μm. **F** The quantification of ThS-stained area (%) (*n* = 6 per group). **G**, **H** Analysis of Aβ_1-40_ **G** and Aβ_1-42_ **H** levels in the hippocampus and cortex using ELISA kits (*n* = 6 per group). Statistical analysis included the Student’s *t*-test. **I** and **J** Images of Iba-1 **I** and GFAP **J** immunostaining in the hippocampus and cortex; scale bar, 100 μm. **K**–**N** The quantification of Iba-1 in the hippocampus **K** and cortex **L** and GFAP in the hippocampus **M** and cortex **N** positive cells area (%) (*n* = 4–6 per group). Statistical analysis included the two-way ANOVA and Tukey’s post hoc test. Bars represent as mean ± standard deviation. Bars of the Fig. 2B represent as mean ± standard error of the mean. ^#^*P* < 0.05, ^##^*P* < 0.01, ^###^*P* < 0.001, and ^####^*P* < 0.0001 *vs. *WT mice. *P < 0.05, **P < 0.01, and ***P < 0.001 *vs. *5xFAD mice

To explore the potential effects of M cell depletion on learning and memory function in 5xFAD mice, we performed the Y-maze and MWM tests. These tests are commonly used to investigate spatial learning and memory function in preclinical studies. In the Y-maze test, 5xFAD/*Spib*^−/−^ mice showed improved memory, as evidenced by a significant increase in the spontaneous alternations (%) compared with 5xFAD mice, without a decrease in the number of total entries, interaction between 5xFAD and *Spib* showed a significant difference (F_1,64_ = 4.396, *P* = 0.0400) (Fig. 2A, Additional file 1: Fig. S5A). In the MWM test, the 5xFAD/*Spib*^−/−^ mice found the hidden platform faster than the 5xFAD mice from day 6 (Fig. 2B). In the probe test on day 11, 5xFAD/*Spib*^−/−^ mice not only crossed the platform as much as the WT mice did, but also stayed in the quadrant where the platform had previously been located without showing differences in swimming speed and total distance, while 5xFAD mice did not (Fig. 2C, D, Additional file 1: Fig. S5B–D). In addition, there was no significant difference in the results between the WT and WT/*Spib*^−/−^ mice. We further investigated whether M cell depletion affects memory functions in late-stage of AD, but there was no significant effect in 9-month-old 5xFAD mice (Additional file 1: Fig. S6A–F).

Next, we analyzed Aβ deposition in the brain to investigate whether memory improvements induced by the inhibition of M cells correlated with the Aβ pathology. First, the frozen brain tissues sections were stained with ThS, which is widely used to detect Aβ plaques. We observed a marked reduction of ThS-positive areas (%) in the brains of 5xFAD/*Spib*^−/−^ mice compared to those in 5xFAD mice (Fig. 2E, F). To further investigate Aβ deposition, we performed immunofluorescence using 6E10 and 4G8 antibodies to detect Aβ_1-16_ and Aβ_17-24_, respectively. The area showing Aβ accumulation was decreased in the brains of 5xFAD/*Spib*^−/−^ mice than in those of 5xFAD mice (Additional file 1: Fig. S7A and B). In addition, an ELISA confirmed that the staining results accurately reflected quantitative changes in brain Aβ protein levels. The brains of the 5xFAD/*Spib*^−/−^ mice had significantly reduced Aβ_1-42_ levels and slightly decreased Aβ_1-40_ levels compared to those in 5xFAD mice (Fig. 2G, H). These effects were not observed in 9-month-old 5xFAD/*Spib*^−/−^ mice (Additional file 1: Fig. S6G–I). These findings suggest that M cell depletion ameliorates memory dysfunction and Aβ deposition in the 6-month-old 5xFAD mice.

### Inhibition of M cells inhibits microglial activation

To determine the mechanisms responsible for the amelioration of Aβ accumulation in 6-month-old 5xFAD/*Spib*^−/−^ mice, we first analyzed the Aβ processing signaling factors such as amyloid precursor protein (APP), PS1, and ADAM10, as well as Aβ degrading enzymes such as NEP and IDE by using Western blotting, but did not detect differences between the 5xFAD and 5xFAD/*Spib*^−/−^ mice (Additional file 1: Fig. S8).

Neuroinflammation is a major feature of AD lesions and is known to promote Aβ generation and accumulation [20]. We stained brain tissues using Iba-1 (a microglial marker) and GFAP (an astrocyte marker) specific antibodies to investigate the changes in reactive gliosis caused by the inhibition of M cells. Although M cell depletion was not affected in GFAP^+^ astrocytes, Iba-1^+^ microglia were markedly decreased in the brains of 5xFAD/*Spib*^−/−^ mice (Fig. 2I–N). Microglia stimulated by Aβ deposition express several pro-inflammatory cytokines, including tumor necrosis factor-α (TNF-α), interleukin-6 (IL-6), and interleukin-1β (IL-1β). We measured the mRNA levels of these proinflammatory cytokines, and found them elevated in the brains of 5xFAD mice compared with those of WT mice. In contrast, inhibition of M cells marginally reduced proinflammatory cytokine levels of *Tnf-α* and *Il-1β* in the hippocampus (interaction between 5xFAD and *Spib* showed a significant difference (*Tnf-α*, F_1,30_ = 4.171, *P* = 0.0500; *Il-1β*, F_1,30_ = 4.501, *P* = 0.0423)), but not in the cortex, of 5xFAD mice (Additional file 1: Fig. S7C–H). Furthermore, to further identify functional reactive states of microglia, we measured mRNA expression levels of *Cx3cr1*, *Ctsd*, *Tyrobp*, and *Cst7*. These factors were slightly decreased in the hippocampus of 5xFAD/*Spib*^−/−^ mice, interaction between 5xFAD and *Spib* showed a significant difference (*Cx3cr1*, F_1,30_ = 7.255, *P* = 0.0115) (Additional file 1: Fig. S7I–L). Overall, these results found that a decline in Aβ accumulation following M cell depletion could be correlated with a decrease in excessive neuroinflammation.

### Inhibition of M cells enhances microglial Aβ phagocytosis

**Fig. 3:**
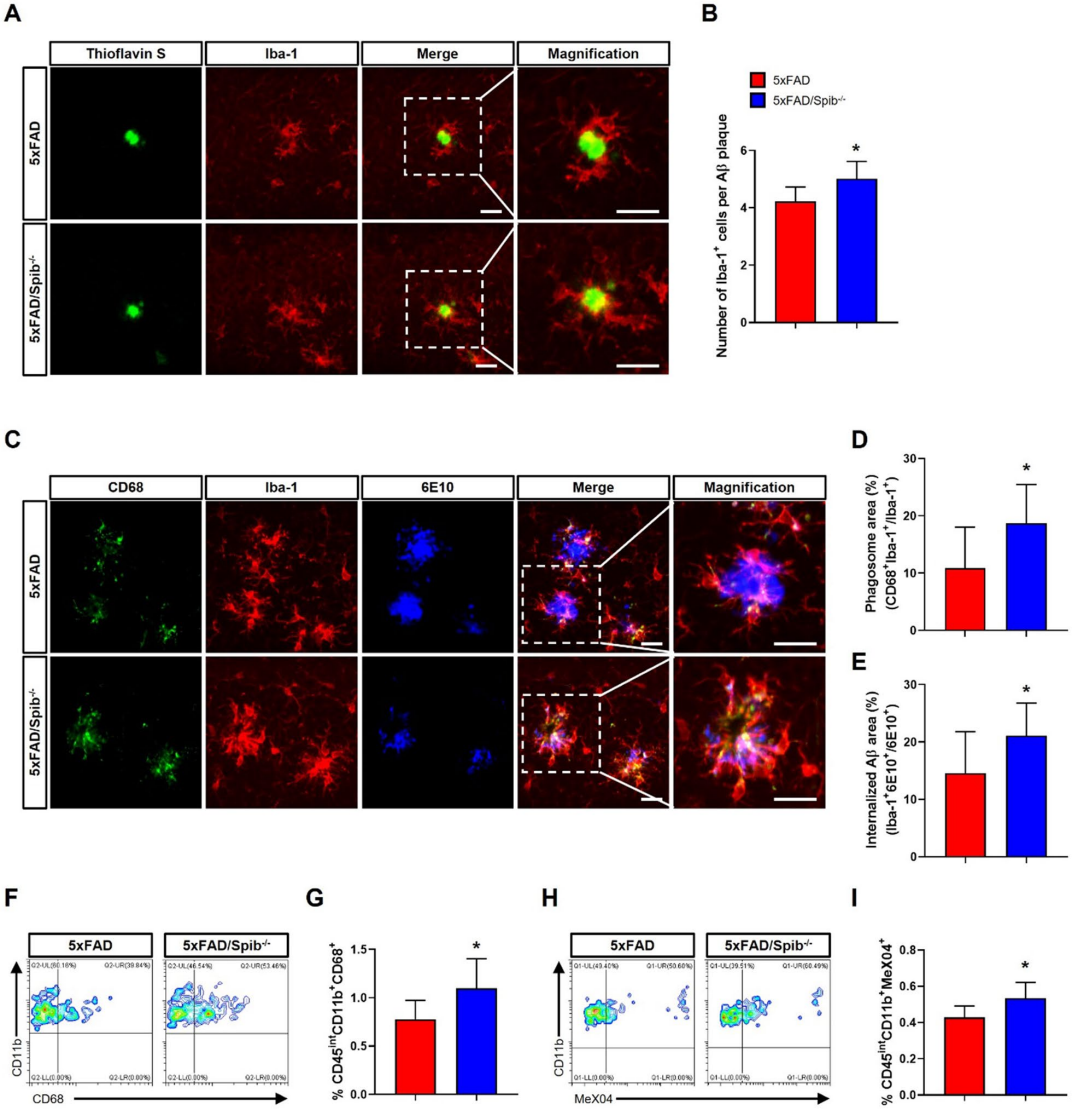
Depletion of microfold cells enhanced microglial phagocytosis in 5xFAD mice. **A** Images of Aβ plaques (thioflavin S, green) and microglia (Iba-1, red); scale bar, 20 μm. **B** The quantification of microglia surrounding Aβ plaques (*n* = 9 per group). **C** Immunofluorescence images of lysosome (CD68, green), microglia (Iba-1, red), and Aβ (6E10, blue); scale bar, 20 μm. **D**, **E** The quantification of phagosome (CD68^+^Iba-1^+^/Iba-1^+^) area (%) **D** and internalized Aβ (Iba-1^+^6E10^+^/6E10^+^) area (%) **E** (*n* = 9 per group). **F**–**I** Flow cytometric analysis of the quantification of and CD68-positive microglia (CD45^int^CD11b^+^) **F** and **G** and MeX04-positive microglia (CD45^int^CD11b^+^) **H** and **I** in the brain (*n* = 8 per group). Bars represent as mean ± standard deviation. Statistical analysis included the Student’s *t*-test. *P < 0.05 *vs. *5xFAD mice

Microglia, the major phagocytic cells of the central nervous system (CNS), are responsible for clearing misfolded proteins such as Aβ. A decline in their function exacerbates AD pathology and increases Aβ deposition. Proinflammatory cytokines, such as TNF-α and IL-1β, reportedly reduce Aβ clearance by microglia [21]. Thus, to determine whether the decrease in Aβ accumulation in M cell-depleted 5xFAD mice is related to the microglial phagocytosis against Aβ, we quantified the number of microglia (Iba-1^+^) surrounding Aβ plaques (ThS^+^). Confocal images showed that the number of microglia around Aβ plaques was increased in 5xFAD/*Spib*^−/−^ mice compared to that in 5xFAD mice, suggesting that an increased number of microglia is involved in Aβ phagocytosis (Fig. 3A, B). To further investigate the Aβ phagocytic aptitude of microglia, we carried out co-immunostaining of CD68, which overexpressed in phagocytic microglia, along with Iba-1 and 6E10 specific antibodies. M cell-depleted 5xFAD mice showed an increased number of microglia containing co-stained lysosomes and Aβ compared to that in 5xFAD mice (Fig. 3C–E).

Additionally, to validate the immunofluorescence results, we analyzed the number of CD68- or Aβ-positive microglia in the brains using flow cytometry. The number of CD68-positive microglia (CD45^int^CD11b^+^) was significantly increased in the brains of 5xFAD/*Spib*^−/−^ mice compared to those in 5xFAD mice (Fig. 3F and G). Next, we injected a fluorescent derivative of MeX04, which crosses the blood–brain barrier and binds to Aβ, into 5xFAD and 5xFAD/*Spib*^−/−^ mice. A nearly-1.5-fold increase in microglial Aβ phagocytosis (CD45^int^CD11b^+^MeX04^+^) was observed in 5xFAD/*Spib*^−/−^ mice compared with 5xFAD mice (Fig. 3H and I). These results demonstrate that the inhibition of M cells increased microglial recruitment surrounding Aβ plaques and promoted microglial Aβ phagocytic function, thereby reducing Aβ accumulation in the brains of 5xFAD mice.

### Inhibition of M cells regulates infiltrated peripheral immune cells in the brain

**Fig. 4:**
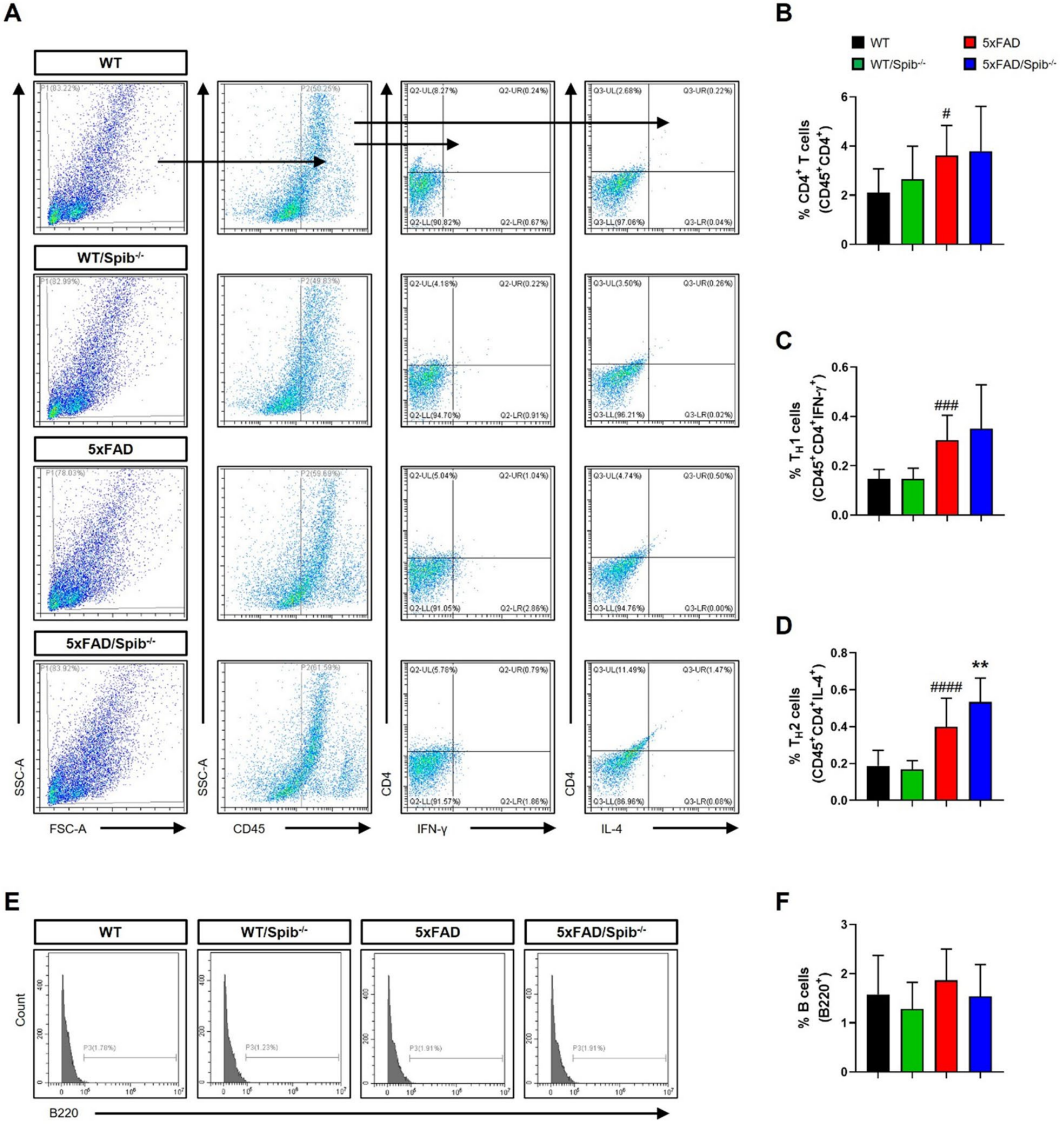
Depletion of microfold cells increased the count of brain infiltrating T_H_2 cells in 5xFAD mice. **A** Gating strategy for detection of brain infiltrating CD45^high^CD4^+^ (CD4^+^ T), CD45^high^CD4^+^IFN-γ^+^ (T_H_1) cells, and CD45^high^CD4^+^IL-4^+^ (T_H_2) cells. **B**–**D** Graph displaying the calculated percentage of infiltrating CD45^high^CD4^+^ (CD4^+^ T) cells **B**, CD45^high^CD4^+^IFN-γ^+^ (T_H_1) cells **C**, and CD45^high^CD4^+^IL-4^+^ (T_H_2) cells **D** in WT, WT/*Spib*^−/−^, 5xFAD, and 5xFAD/*Spib*^−/−^ mouse brains (*n* = 13–17 per group). **E** Histograms are representative of B220^+^ B cells at the brain. **F** Graph displaying the calculated percentage of B220^+^ B cells in WT, WT/*Spib*^−/−^, 5xFAD, and 5xFAD/*Spib*^−/−^ brains (*n* = 4–6 per group). Bars represent as mean ± standard deviation. Statistical analysis included the two-way ANOVA and Tukey’s post hoc test. ^#^*P* < 0.05, ^###^*P* < 0.001, and ^####^*P* < 0.0001 *vs. *WT mice. **P < 0.01 *vs. *5xFAD mice

Peripheral immune cells can migrate to other tissues, including the brain, and participate in inflammatory responses of the brain [22]. Since T cells frequently penetrate the AD brain and induce excessive inflammatory response compared to the healthy brain [23], we examined the number of infiltrating CD4^+^ T cells in the brains of WT, WT/*Spib*^−/−^, 5xFAD, and 5xFAD/*Spib*^−/−^ mice using flow cytometry (Fig. 4A). The 5xFAD mice showed an increased infiltration of CD4^+^ T cells into the brain compared with WT mice, similar to the results of a previous study [17], and there was no significant difference between 5 and 5xFAD/*Spib*^−/−^ mice (Fig. 4B). We further examined infiltrating T helper 1 (T_H_1; CD45^high^CD4^+^INF-γ^+^) and T_H_2 (CD45^high^CD4^+^IL-4^+^) cells to analyze the changes in CD4^+^ T cell subtypes. The count of T_H_1 cells was comparable between 5 and 5xFAD/*Spib*^−/−^ mice, but T_H_2 cell counts was significantly increased in 5xFAD/*Spib*^−/−^ mice compared to 5xFAD mice, interaction between 5xFAD and *Spib* showed a significant difference (F_1,58_ = 7.440, *P* = 0.0084) (Fig. 4C and D). This suggested that an increase in T_H_2 cell counts due to M cell deficiency affected microglial phagocytosis. Next, we analyzed B220^+^ B cells as *Spib* is also related to B cells [24]. We did not detect any changes in the number of B220^+^ B cells in the brain following *Spib* knockout (Fig. 4E, F).

### Inhibition of M cells alters the composition of gut microbiota

**Fig. 5:**
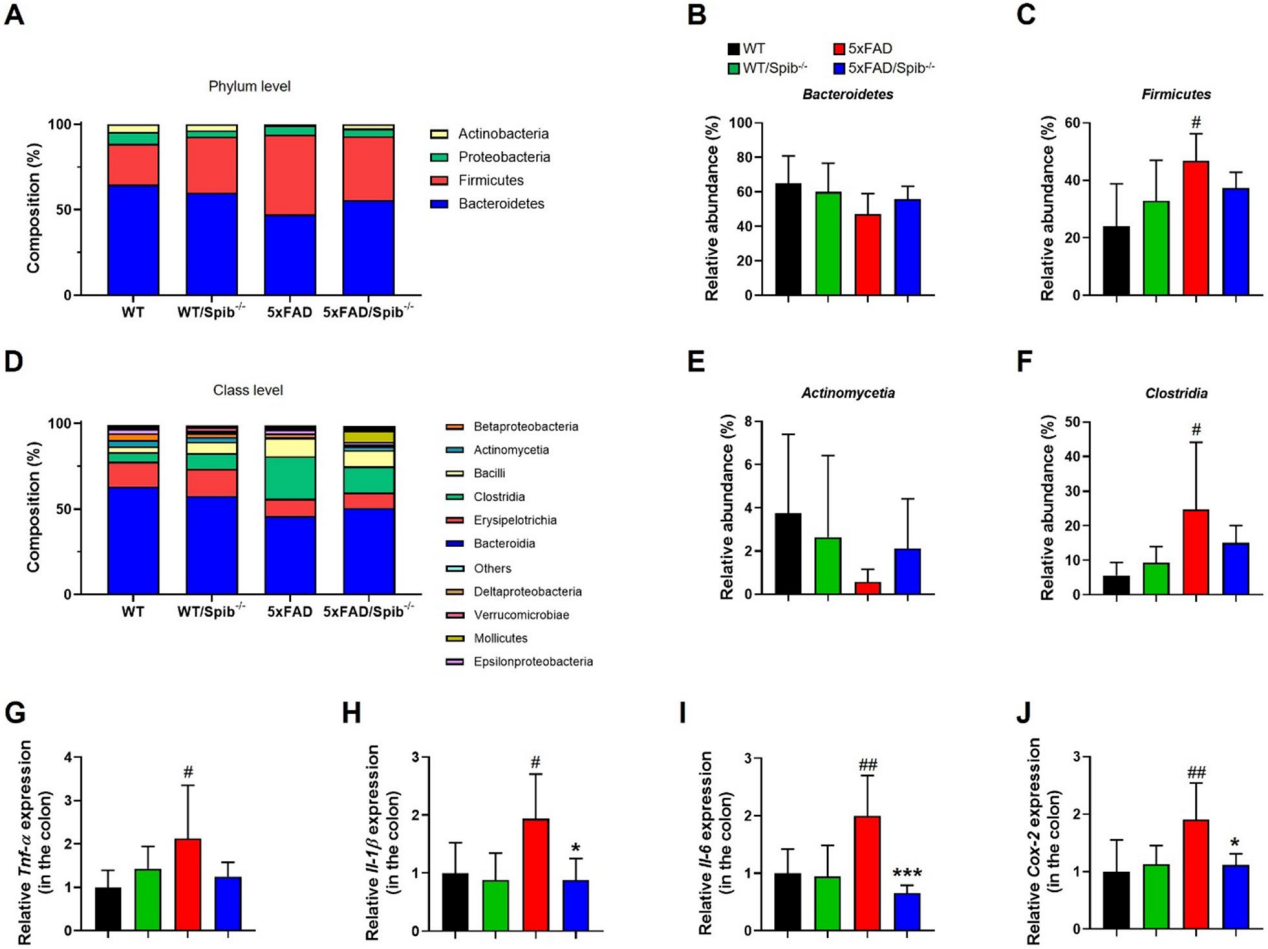
Depletion of microfold cells altered the composition of gut microbiota in 5xFAD mice. **A** and **D** The distribution of the relative abundance of gut microbiota at the phylum **A** and class **D** levels was observed in the four groups (*n* = 5 per group). **B**, **C** Relative abundance of *Bacteroidetes* **B** and *Firmicutes* **C** at the phylum level. **E** and **F** Relative abundance of *Actinomycetia* **E** and *Clostridia* **F** at the class level. **G–J** The gene expression of colonic inflammatory mediators was measured with qRT-PCR (*n* = 6–8 per group). Bars represent as mean ± standard deviation. Statistical analysis included the two-way ANOVA and Tukey’s post hoc test. ^#^*P* < 0.05 and ^##^*P* < 0.01 *vs. *WT mice. *P < 0.05 and ***P < 0.001 *vs. *5xFAD mice

Gut microbiota can control the immune system, which is considered an important regulator of the MGB axis. A previous study showed a link between changes in the gut microbial composition, the peripheral immune system, and the development of AD pathology [25]. To identify whether fecal microbiota was altered in response to M cell deficiency, total DNA was purified from the fecal microbiota of five male 6-months-old mice belonging to the four groups. The diversity of the fecal microbiome were not significantly different between the groups (Additional file 1: Fig. S9). We further identified microbiota abundance at the phylum and class levels (averaged > 1% of the relative abundance) to assess the potential bacterial groups responsible for gut dysbiosis (Fig. 5A–F). At the phylum level, the gut microbiota of 5xFAD/*Spib*^−/−^ mice was similar to that of WT mice but different from that of 5xFAD mice (Fig. 5A–C). At the class level, 5xFAD/*Spib*^−/−^ mice showed an increased numbers of *Actinomycetia*, which are anti-inflammatory and antioxidant bacteria, compared with 5xFAD mice (Fig. 5E). The proportion of microbiota belong to the class *Clostridia* (the inflammatory bacteria) increased in 5xFAD mice but decreased in 5xFAD/*Spib*^−/−^ mice (Fig. 5F). These analyses revealed that the inhibition of M cells influenced the composition of the gut microbiota in the 5xFAD mice.

Since proinflammatory cytokines are released during gut infections and dysbiosis and the count of inflammatory bacteria were reduced in 5xFAD/*Spib*^−/−^ mice, we investigated the mRNA levels of proinflammatory cytokines in the colon of these mice. The mRNA levels of proinflammatory mediators (*Tnf-α*, *Il-6*, *Il-1β*, and *Cox-2*) were elevated in the colon of 5xFAD mice, but the inhibition of M cells mitigated this phenomenon, interaction between 5xFAD and *Spib* showed a significant difference (*Tnf-α*, F_1,23_ = 6.109, *P* = 0.0213; *Il-6*, F_1,23_ = 4.835, *P* = 0.0382; *Il-1β*, F_1,23_ = 11.39, *P* = 0.0026; *Cox-2*, F_1,23_ = 6.415, *P* = 0.0186) (Fig. 5G–J). Collectively, these results illustrate that the M cell blockade can affect the brain microenvironments through changes caused in gut, including microbiota composition and peripheral immune systems.

### Inhibition of M cells blocks AD pathology induced by 5xFAD-derived microbiota

**Fig. 6:**
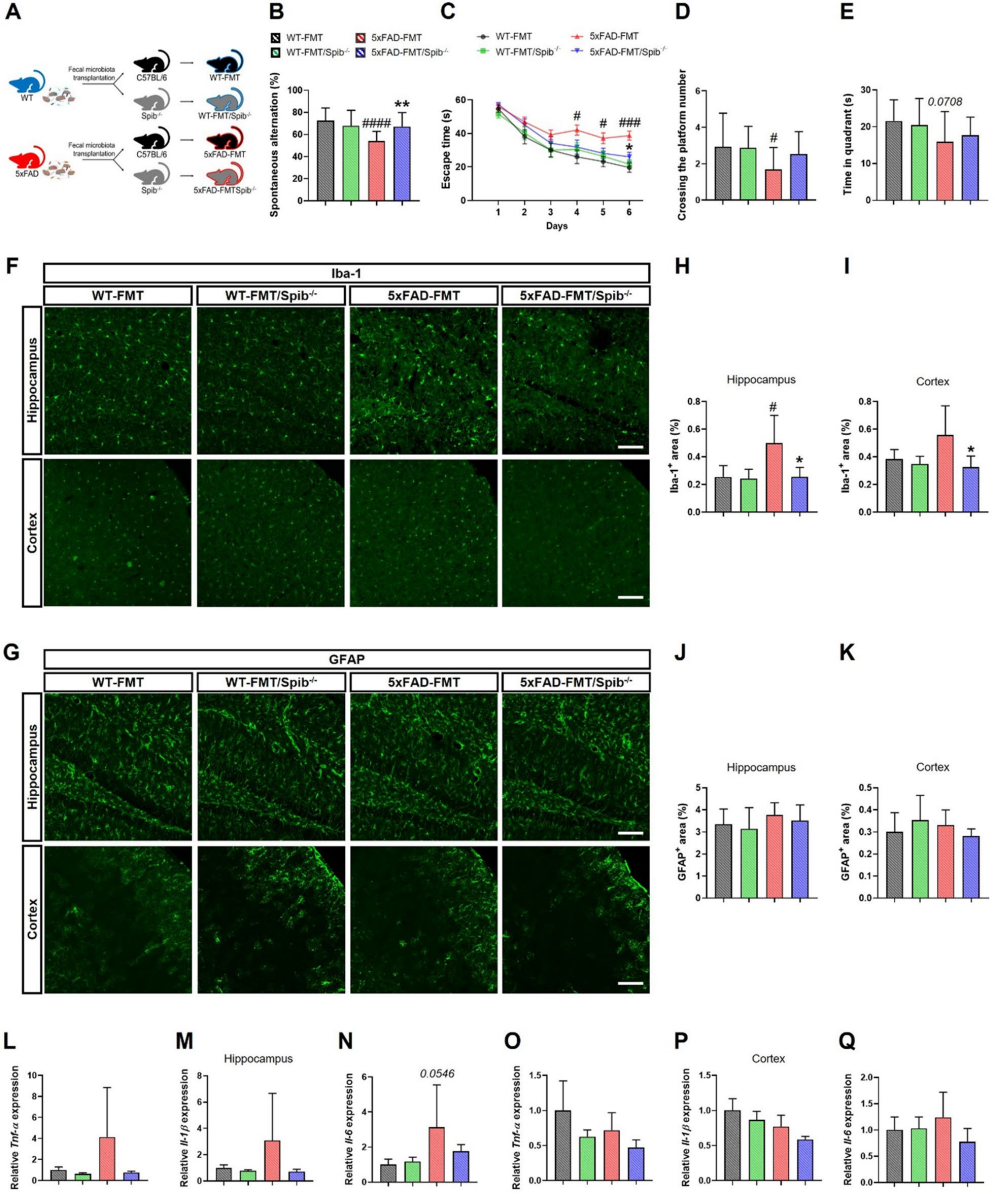
The effect of 5xFAD-fecal microbiota transplantation (FMT) on behavioral changes and neuroinflammation in *Spib*^−/−^ mice. **A** Experimental design **B** Spontaneous alternations (%) of WT-FMT, WT-FMT/*Spib*^−/−^, 5xFAD-FMT, and 5xFAD-FMT/*Spib*^−/−^ mice (WT-FMT, *n* = 16; WT-FMT/*Spib*^−/−^, *n* = 15; 5xFAD-FMT, *n* = 19; and 5xFAD-FMT/*Spib*^−/−^ mice, *n* = 17). **C** Escape latencies of WT-FMT, WT-FMT/*Spib*^−/−^, 5xFAD-FMT, and 5xFAD-FMT/*Spib*^−/−^ mice over 6 days. **D**, **E** In the probe trial on day 7, crossing the platform number **D** and spending time in the target quadrant **E** were recorded and analyzed. **F**, **G** Immunofluorescence images of Iba-1 **F** and GFAP **G** staining in the hippocampus and cortex; scale bar, 100 μm. **H**–**K** The quantification of Iba-1 in the hippocampus **H** and cortex **I** and GFAP in the hippocampus **J** and cortex **K** positive cells area (%) (*n* = 4–6 per group). **L**–**Q** The inflammatory cytokines were measured in the hippocampus **L**–**N** and cortex **O**–**Q** using qRT-PCR (*n* = 4–5 per group). Bars represent as mean ± standard deviation. Bars of the Fig. 6C represent as mean ± standard error of the mean. Statistical analysis included the two-way ANOVA and Tukey’s post hoc test. ^#^*P* < 0.05, ^###^*P* < 0.001 and ^####^*P* < 0.0001 *vs. *WT-FMT mice. *P < 0.05 and **P < 0.01 *vs. *5xFAD-FMT mice

As shown in Fig. 1, the 5xFAD-derived microbiota can increase the number of M cells in the colon. We previously demonstrated that 5xFAD-derived microbiota can cause memory impairment and colonic inflammation [7]. Based on these findings, we hypothesized that memory deficits caused by the 5xFAD-derived microbiota would not be induced in M cell-deficient mice. In line with previous results, 5xFAD-FMT caused memory dysfunction in naïve mice. However, in *Spib*^−/−^ mice, 5xFAD-FMT failed to impair the learning and memory functions in the Y-maze and MWM tests, interaction between 5xFAD-FMT and *Spib* showed a significant difference (Y-maze, F_1,63_ = 9.634, *P* = 0.0029) (Fig. 6B–E). Furthermore, the M cell-depleted mice showed no significant changes in locomotor activity (Additional file 1: Fig. S10).

To determine whether the inhibition of M cells suppressed changes in the brain microenvironment caused by 5xFAD-FMT, we examine the extent of neuroinflammation. An analysis of reactive gliosis using immunofluorescence showed that the number of GFAP^+^ astrocytes did not change in 5xFAD-FMT mice, consistent with the previous results. The count of Iba-1^+^ microglia was increased in the brains of 5xFAD-FMT mice but not in 5xFAD-FMT/*Spib*^−/−^ mice (Fig. 6F–K). Moreover, in the brains of *Spib*^−/−^ mice, the expression of pro-inflammatory cytokines (*Tnf-α*, *Il-6*, and *Il-1β*) did not increase following exposure to the 5xFAD-derived microbiota (Fig. 6L–Q). The mRNA levels of these cytokines in the brains of 5xFAD-FMT/*Spib*^−/−^ mice were similar to those in WT-FMT and WT-FMT/*Spib*^−/−^ mice. The results from 5xFAD-derived microbiota-treated *Spib*^−/−^ mice evidenced that the inhibition of M cells may prevent AD pathology, including memory deficits and excessive neuroinflammation mediated by 5xFAD-derived gut microbiota.

## Discussion

In this study, we demonstrated, for the first time, that M cell counts were increased in the PPs and/or colon of AD-mimicked mice, including 5xFAD and 5xFAD-FMT mice. The downregulation of M cells further ameliorated Aβ accumulation and rescued cognitive dysfunction. In addition, we found T_H_2 cell-mediated restoration of microglial function in the M cell-depleted 5xFAD mice. Moreover, gut dysbiosis of 5xFAD mice was restored by inhibition of M cell formation, which was similar to gut microbiota composition of WT mice. We also found that 5xFAD mice-derived microbiota caused memory impairments in naïve mice, but not in M cell-depleted mice. These results provide strong evidence that M cells may be a therapeutic target for AD.

Gut microbiota dysbiosis is considered a risk factor for CNS diseases including AD [3]. Accumulating evidence from clinical studies has shown that the fecal microbiota composition in patients with AD differs from that in healthy humans. *E. coli* K99 pili protein and LPS were observed in the brain of AD patients, and LPS and Aβ are colocalized in vessel walls [26, 27]. The stool of Aβ-positive patients with cognitive impairment had an increased proportion of *Escherichia*/*Shigella* and *Bacteroides fragilis* compared with that of Aβ-negative patients with cognitive impairment and healthy controls [28]. Vogt et al. reported reduced levels of *Firmicutes* and increased levels of *Bacteroidetes* in the fecal microbiota of patients with AD compared to healthy controls [29]. In contrast, Zhuang and colleagues showed that the proportion of *Firmicutes* increased, whereas that of *Bacteroidetes* decreased in the feces of AD patients compared to the age-matched healthy controls [30]. Differences in gut microbiota composition have been reported in WT and AD transgenic mice; some studies showed changes similar to clinical results, while other studies reported opposite results [31–34]. These discrepancies can arise from different experimental settings, including the sex, age, strain of the mice used. We confirmed gut dysbiosis in 6-month-old 5xFAD mice, and our results were similar to those observed for patients with ulcerative colitis [35]. However, the inhibition of M cells in 5xFAD mice altered the abundance of fecal microbiota, similar to that observed in WT mice. This suggests that the density of M cells correlates with the composition of the gut microbiome, which may even affect the brain microenvironments.

Typical M cells, which play a pivotal role in the immune surveillance of mucosal tissues, are dedicated to detecting external stimuli, including pathogenic microbiota. On the other hand, there are distinct M cells that exist in absence of lymphoid tissues, and can form in inflamed gut condition [36]. The number of M cells is increased in experimental colitis and in the inflamed gut of humans with ulcerative colitis [16, 18]. Furthermore, indomethacin-induced intestinal inflammation increases formation of M cells [37]. Parnell et al. reported that TNF-α and TNF receptor 2 are required for generating inducible colonic M cells, whereas constitutive M cells are dependent on other triggers, including *Rankl* [38]. In this study, we observed an increase in the mRNA levels of *Tnf-α* and *Rankl*, as well as GP2-positive cell counts in the colons of 5xFAD and 5xFAD-FMT mice, suggesting that M cell counts were increased in the AD microenvironments. Further studies are required to identify the gut bacteria and its mechanisms that induce the formation of colonic M cells in the AD environments.

All types of M cells can be used by pathogenic microbiota and proteins to invade the host. *Candida albicans* and *E. coli* can employ M cells as a gateway to enter through the intestinal barrier [12, 13]. *Salmonella typhimurium* can transform epithelial cells into M cells by activating the epithelial-mesenchymal transition pathway with an effector protein called SopB. It can then penetrate the intestines via M cells [15]. In this regard, there have been attempts to prevent the onset of disease by blocking the invasion of foreign antigens through M cells. *Mycobacterium tuberculosis* translocates across M cells to initiate infection in vivo, but the genetic inhibition of *Spib* or IK22-5 (RANKL monoclonal antibody) treatment prevents *M. tuberculosis* dissemination, leading to improved survival of mice [19]. The intraperitoneal injection of botulinum neurotoxin is lethal in all mice, including M cell-depleted mice; however, susceptibility to intragastrically administered botulinum neurotoxin is significantly decreased in the *Gp2*^−/−^ or RANKL antibody-treated mice [39]. Prion protein, a substance that causes spongiform encephalopathy, can be delivered to the CNS through the peripheral nervous system. However, the inhibition of M cell induction using RANKL antibody prevents prion protein accumulation in the brain and gut as well as gliosis activation [14]. Notably, gut-innervating nociceptor sensory neurons downregulate the density of M cells by releasing calcitonin gene-related peptide to promote host defense against Salmonella infections [40]. These findings delineate that the increased the invasion of pathogenic antigens through M cells can worsen disease pathology, thus inhibiting M cell induction can prevent this phenomenon. Indeed, there would be difficulties in M cell depletion to be practically carried out in AD patients. To suggest practical treatment to manipulate M cell-mediated pathological aggravation of AD, studies would be required on (1) antibiotics that would eliminate specific M cell-inducing microorganisms; and (2) small molecules that would restrain M cell-inducing molecular pathways. We revealed that AD symptoms, including Aβ deposition and memory dysfunction, were attenuated in M cell-depleted 5xFAD mice at 6-month-old but not in 5xFAD mice at 9-month-old. Gut microenvironment is exacerbated in AD mouse models in an age-dependent manner [10]. We presume that the ineffectiveness of M cell inhibition on AD lesions in 9-month-old 5xFAD mice was due to leaky gut because harmful bacteria, metabolite, and toxins invade into body via disrupted gut barrier [41]. In addition, 5xFAD mice not only have gut dysbiosis but also excessive accumulation of Aβ in the brain as they age, so it may be difficult to alleviate AD lesions only by controlling the intestinal environment. Nevertheless, this study is the first to demonstrate that the regulation of M cell density can influence the reduction of AD pathogenesis. Further studies are needed to elucidate the age-dependent M cell function in AD environments.

In the CNS, microglia are effective phagocytes for misfolded proteins, such as Aβ. Microglia express CX3CR1, a receptor that regulates the activity and migration of these cells. Although CX3CL1/CX3CR1 signaling commonly plays a role associated with homeostatic microglia, this signal is controversial in Aβ pathologies [42]. Nevertheless, studies have reported increased microglial phagocytosis and reduced Aβ deposition in Cx3CR1-deficient AD mouse models [43, 44]. Microglia also can be controlled by intestinal microbiota [45]. Germ-free 5xFAD mice (4-month-old) manifested increased microglial Aβ clearance, leading to restored Aβ deposition and memory deficits [11]. The transfer of healthy microbiota reduces Aβ accumulation and microglial activation via modulating gut microbiota along with the intestinal and systemic inflammation in transgenic AD mouse models showing Aβ deposition and neurofibrillary tangles [46]. This suggests that gut microbiota can influence Aβ pathology by modulating microglia.

Although the mechanistic association between gut dysbiosis and microglia in AD is not fully understood, peripheral immune cells have been reported to influence in the brain microenvironments. In the brains of AD transgenic mice, an increase in infiltrating T_H_1 cell counts and a decrease in infiltrating T_H_2 cell counts was observed with aging [47]. Wang et al. demonstrated that decreasing the number of infiltrating T_H_1 cells by remodeling the gut microbiota improved microglial phagocytic capacity, resulting in decreased Aβ pathology [47]. The transfer of Aβ-specific T_H_1 cells into APP/PS1 mice exacerbated AD pathology, including microglial activation, Aβ accumulation, and memory dysfunction [48]. In this study, the inhibition of M cells did not cause any changes in infiltrating T_H_1 (CD45^high^CD4^+^IFN-γ^+^) cell counts in the brains of 5xFAD mice, but it increased infiltrating T_H_2 (CD45^high^CD4^+^IL-4^+^) cell counts. Despite the role of T_H_2 cells is not clearly understood, several studies have reported that T_H_2 cells are involved in the reduction of Aβ. For example, immunization with Aβ_1-42_ in the APP transgenic mice enhanced T_H_2 cell counts and decreased T_H_1 cell counts in the spleen, thereby mitigating the Aβ burden [49]. The infusion of Aβ-specific T_H_2 cells not only suppressed microglial activation and Aβ deposition, but also restored cognitive function in the APP/PS1 mice [50]. Microglia are activated by Aβ-specific T_H_1 cell stimulation, whereas Aβ-specific T_H_2 cells reverse this effect [51]. These findings suggest that an increase in T_H_2 cell number in the brain prevents AD symptoms induced by Aβ deposition and microglial activation. Although the mechanism by which T_H_2 cell numbers are increased is not yet well-known, we thought that the gut microbiota plays an important role in this process. Further studies should be conducted to determine the association of the bacteria with the increase in T_H_2 cells in the brain.

## Conclusions

In conclusion, we investigated the role of M cells against AD pathology. We demonstrated that the depletion of M cells in the AD microenvironments changes the composition of the gut microbiota, thereby attenuating AD pathological features including memory dysfunction, Aβ accumulation, excessive neuroinflammation, and immune response. Our findings suggest that M cells play a crucial role in AD progression based on the MGB axis. Therefore, the regulation of M cell density (through practical methods, e.g., antagonizing M cell-inducing microorganisms and controlling gut epithelial cell signaling) may be a novel and potential therapeutic strategy for microbiota-aggravated neurodegenerative diseases, including AD.

### Supplementary Information

**Additional file 1:** **Figure S1.** Changes in microfold cells in the Peyer’s patches (PPs) of 5xFAD and 5xFAD-FMT mice. **Figure S2.** Tight junctions are not reduced in the colons of 5xFAD and 5xFAD-FMT mice. **Figure S3.** Intestinal epithelial cells of the secretory lineage develop normally in AD-mimicked mice. **Figure S4.** Experimental animals at 6 months did not reduce body weight. **Figure S5.** Experimental animals at 6 months did not reduce locomotor activity. **Figure S6.** Experimental animals at 9 months did not affect the behavioral function and Aβ accumulation. **Figure S7.** The effect of M cell depletion on Aβ deposition and neuroinflammation in 5xFAD/*Spib*^-/-^ mice. **Figure S8.** Inhibition of microfold cells did not affect the APP pathway. **Figure S9.** Inhibition of microfold cells did not affect the diversity of gut microbiota in 5xFAD mice. **Figure S10.** *Spib*^-/-^ mice did not reduce locomotor activity. **Table S1.** Diet information.** Table S2.** Primer sequences.

## Data Availability

All data that support this paper are present within the paper and/or the Supplementary Materials. The original datasets are also available from the corresponding author upon request.
