# Supplementary material for: Inhibition of microfold cells ameliorates early pathological phenotypes by modulating microglial functions in Alzheimer’s disease mouse model
Source: J Neuroinflammation. 2023 Nov 27;20:282. doi: 10.1186/s12974-023-02966-9 (PMC10680211; doi:10.1186/s12974-023-02966-9)
Supplement: Supplementary file 1 — Additional file 1: Figure S1. Changes in microfold cells in the Peyer’s patches (PPs) of 5xFAD and 5xFAD-FMT mice. Figure S2. Tight junctions are not reduced in the colons of 5xFAD and 5xFAD-FMT mice. Figure S3. Intestinal epithelial cells of the secretory lineage develop normally in AD-mimicked mice. Figure S4. Experimental animals at 6 months did not reduce body weight. Figure S5. Experimental animals at 6 months did not reduce locomotor activity. Figure S6. Experimental animals at 9 months did not affect the behavioral function and Aβ accumulation. Figure S7. The effect of M cell depletion on Aβ deposition and neuroinflammation in 5xFAD/Spib-/- mice. Figure S8. Inhibition of microfold cells did not affect the APP pathway. Figure S9. Inhibition of microfold cells did not affect the diversity of gut microbiota in 5xFAD mice. Figure S10. Spib-/- mice did not reduce locomotor activity. Table S1. Diet information. Table S2. Primer sequences. [file 12974_2023_2966_MOESM1_ESM.docx]

**Additional file 1: figures and tables**


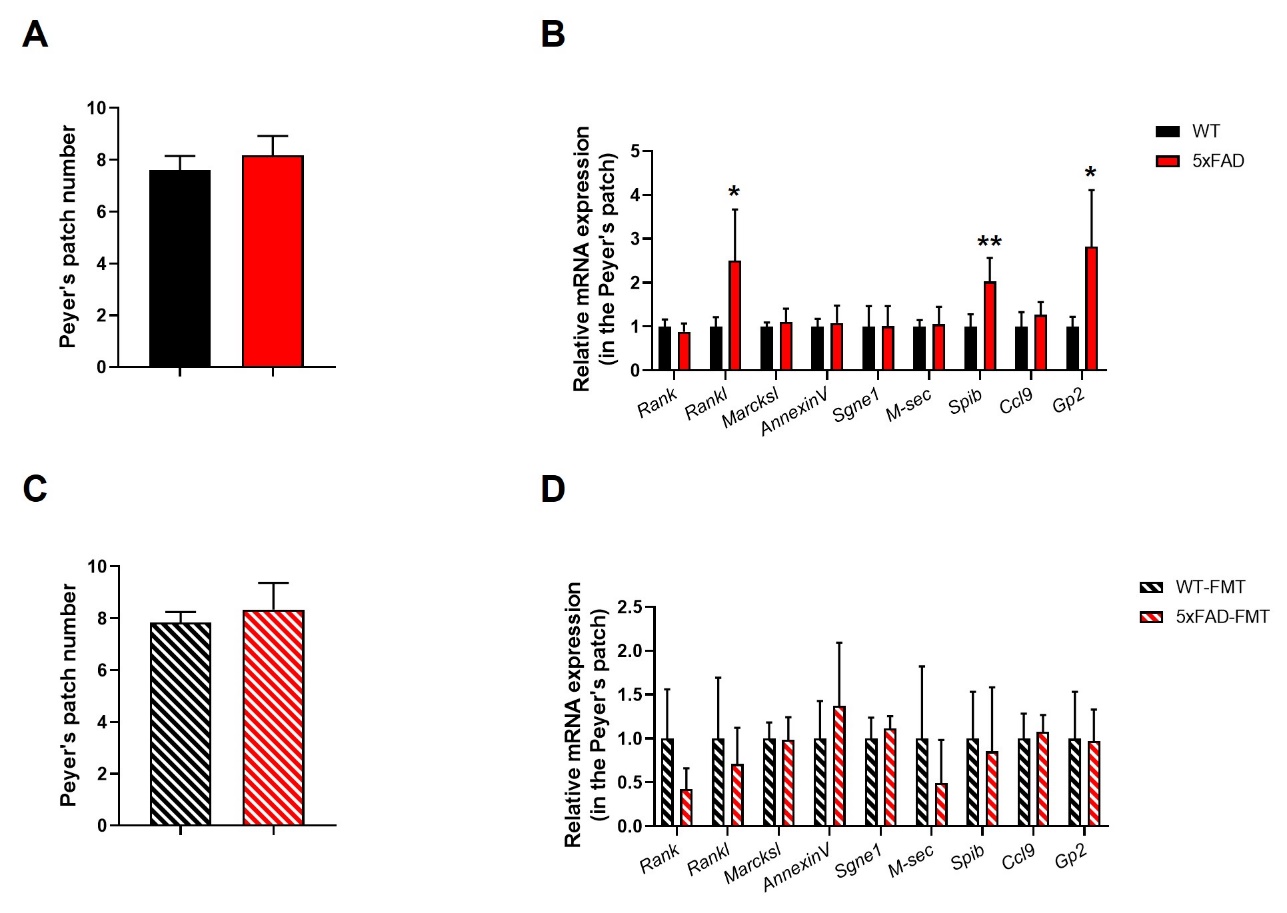


**Figure S1.** Changes in microfold cells in the Peyer’s patches (PPs) of 5xFAD and 5xFAD-FMT mice. (A and C) Number of PPs in the 5xFAD (A) and 5xFAD-FMT (C) mice (*n* = 4–6 per group). (B and D) M cell-related genes in the PPs in 5xFAD (B) and 5xFAD-FMT (D) mice (*n* = 4–6 per group). Bars represent the mean ± standard deviation. Statistical analysis included the Student’s *t*-test. **P* < 0.05 and ***P* < 0.01 *versus* WT mice.


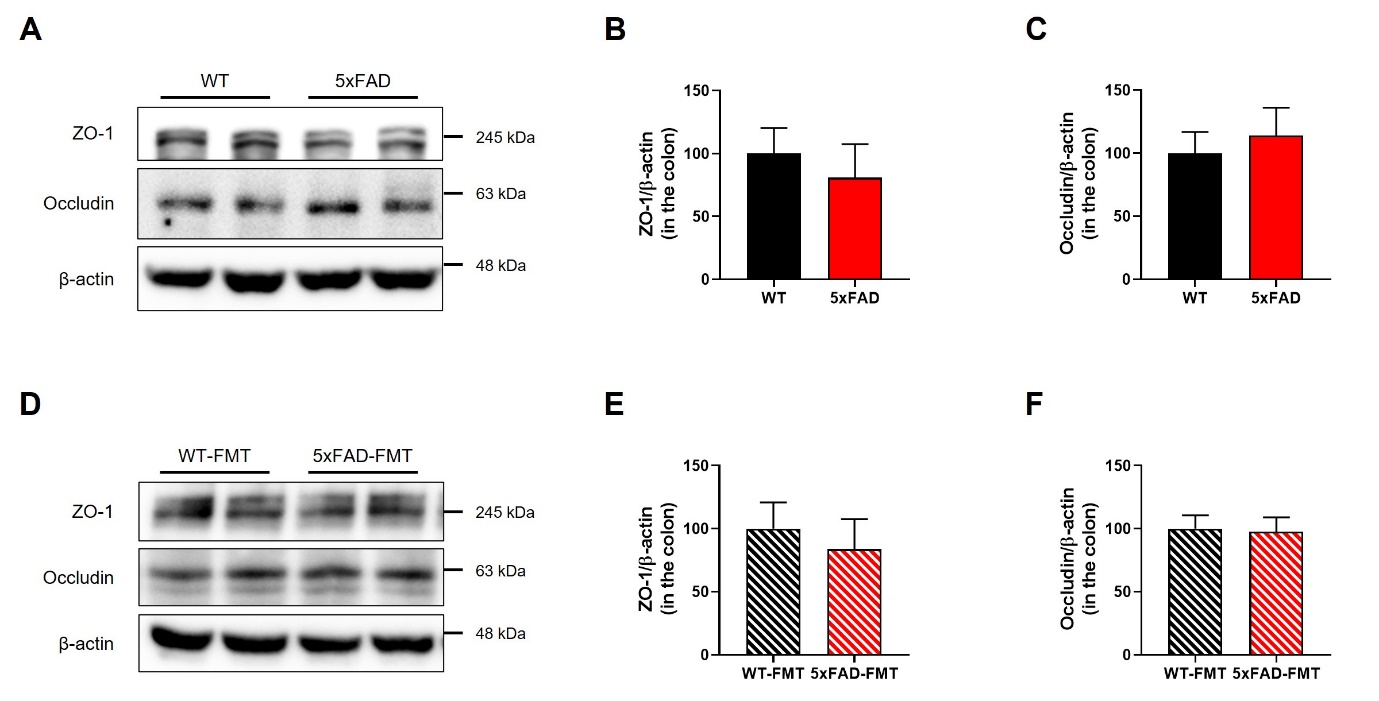


**Figure S2.** Tight junctions are not reduced in the colons of 5xFAD and 5xFAD-FMT mice. (A–F) The expression of ZO-1 and occludin protein was determined in the colon of WT and 5xFAD (A–C) and WT-FMT and 5xFAD-FMT (D–F) by western blotting (*n* = 5–6 per group). Bars represent the mean ± standard deviation. Statistical analysis included the Student’s *t*-test.


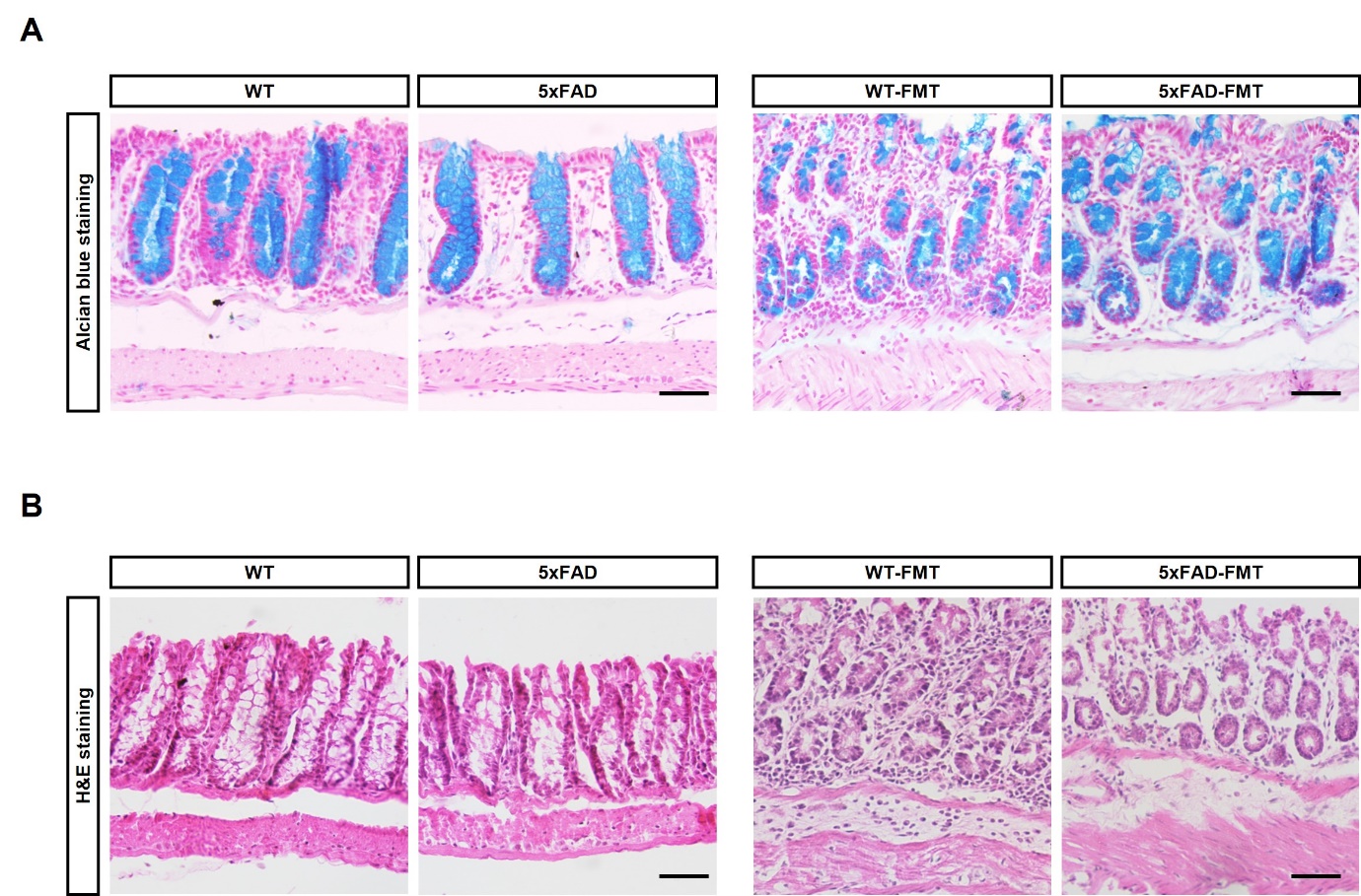


**Figure S3.** Intestinal epithelial cells of the secretory lineage develop normally in AD-mimicked mice. (A) Alcian blue staining was performed to observe the distribution of goblet cells in the colon. (B) For the detection of Paneth cells, colon tissues were stained with H&E. Scale bar, 50 μm. Data are representative of two independent experiments.


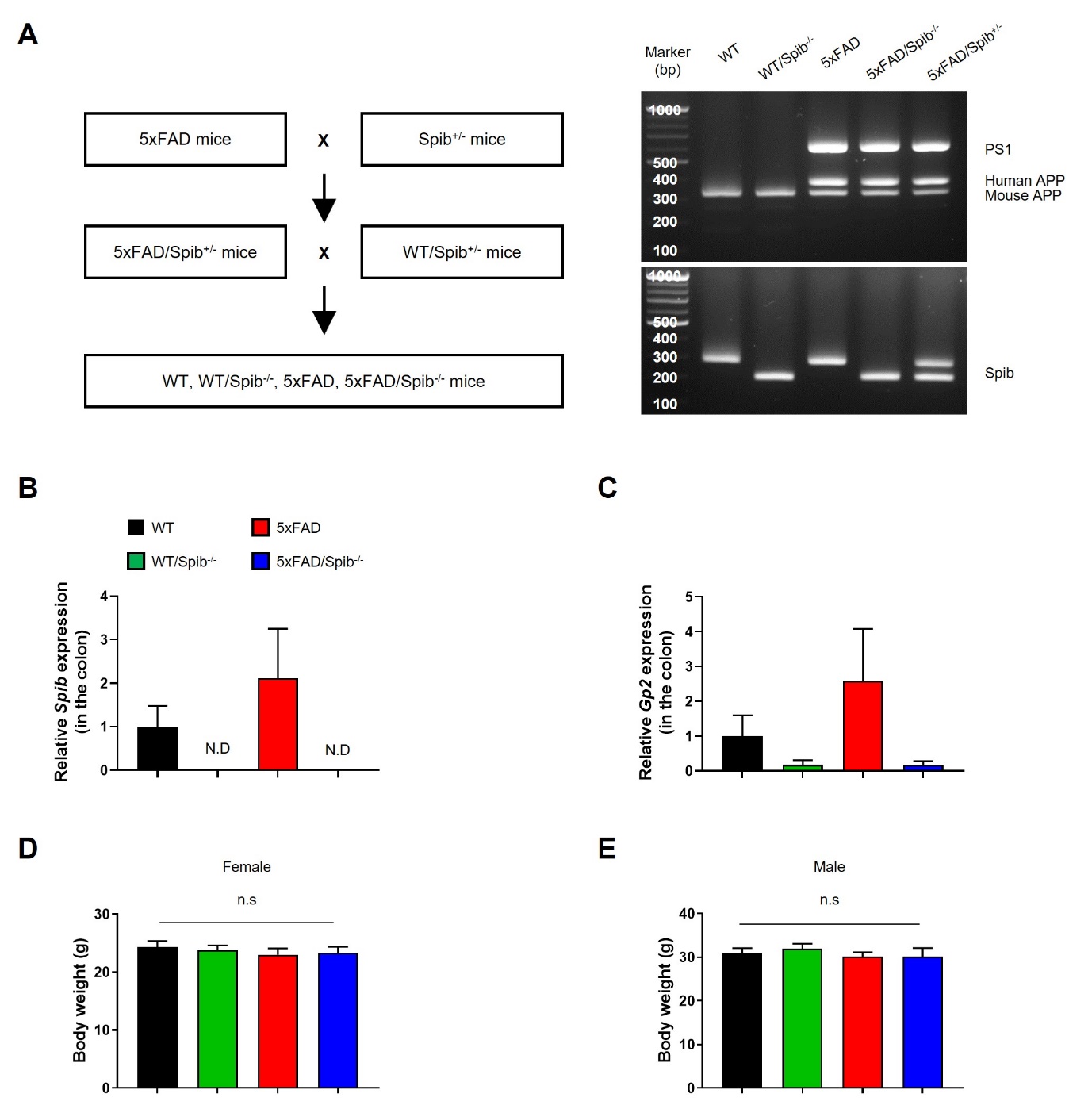


**Figure S4.** Experimental animals at 6 months did not reduce body weight. (A) Mating strategy of the four types of mice. Representative result of genotyping of the five types of mice; WT, WT/*Spib*^-/-^, 5xFAD, 5xFAD/*Spib*^-/-^, 5xFAD/*Spib*^+/-^ mice. (B and C) *Spib* (B) and *Gp2* (C) gene were rarely expressed in the colon of *Spib*^-/-^ mice (*n* = 5–6 per group). (D and E) There was no change in body weight due to *Spib* gene deficiency in both female (D) and male (E) mice (*n* = 5–6 per group). Bars represent the mean ± standard deviation. Statistical analysis included the two-way ANOVA and Tukey’s *post hoc* test. n.s: nonsignificant, N.D: not detected.


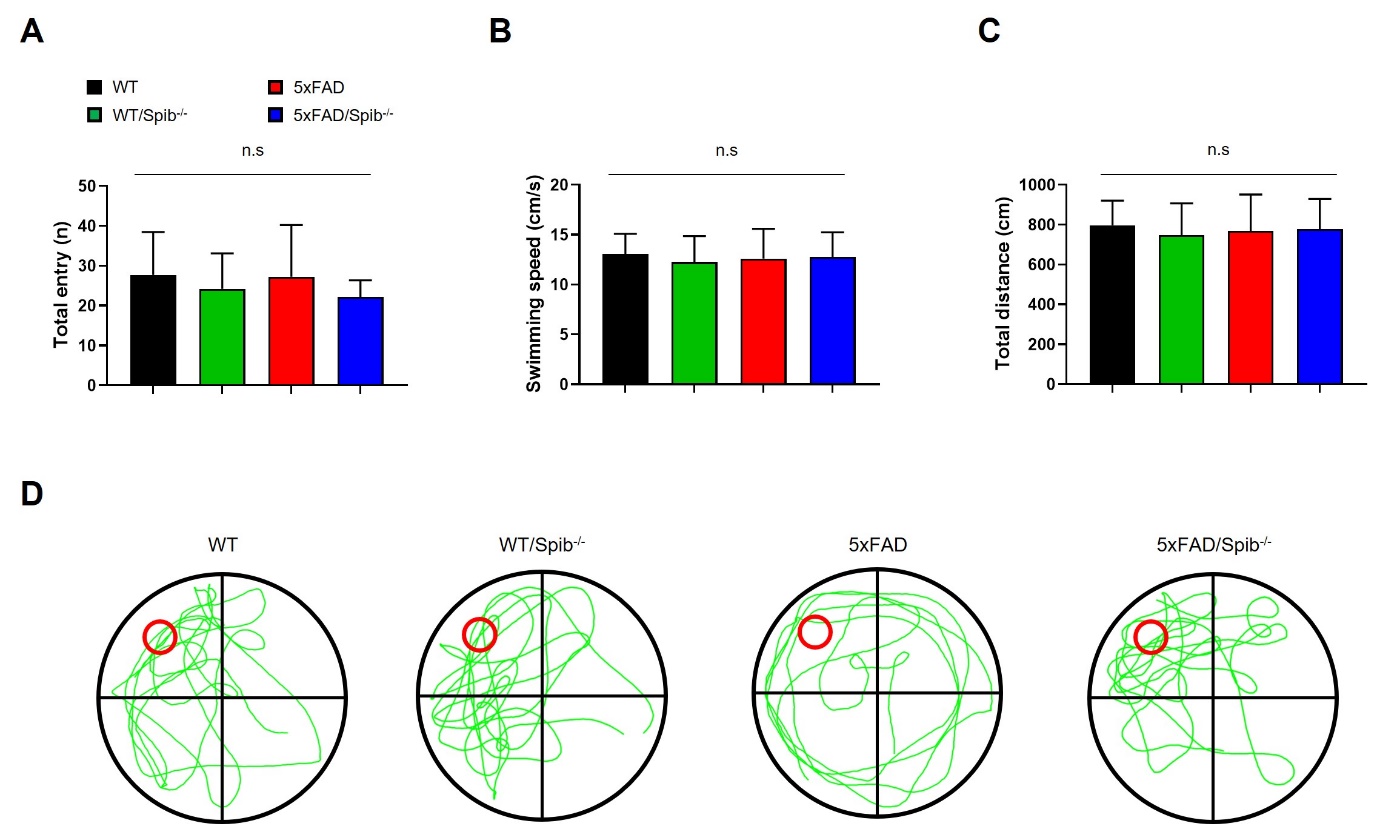


**Figure S5.** Experimental animals at 6 months did not reduce locomotor activity. (A) Total entry in Y-maze test of WT, WT/*Spib*^-/-^, 5xFAD, and 5xFAD/*Spib*^-/-^ mice (WT, *n* = 17; WT/*Spib*^-/-^, *n* = 17; 5xFAD, *n* = 18; 5xFAD/*Spib*^-/-^ mice, *n* = 16). (B and C) On day 11 of the probe trial, swimming speed (B) and total distance (C) of WT, WT/*Spib*^-/-^, 5xFAD, and 5xFAD/*Spib*^-/-^ mice. (D) Representative swimming paths on day 11 of training. Bars represent the mean ± standard deviation. Statistical analysis included the two-way ANOVA and Tukey’s *post hoc* test. n.s: nonsignificant.


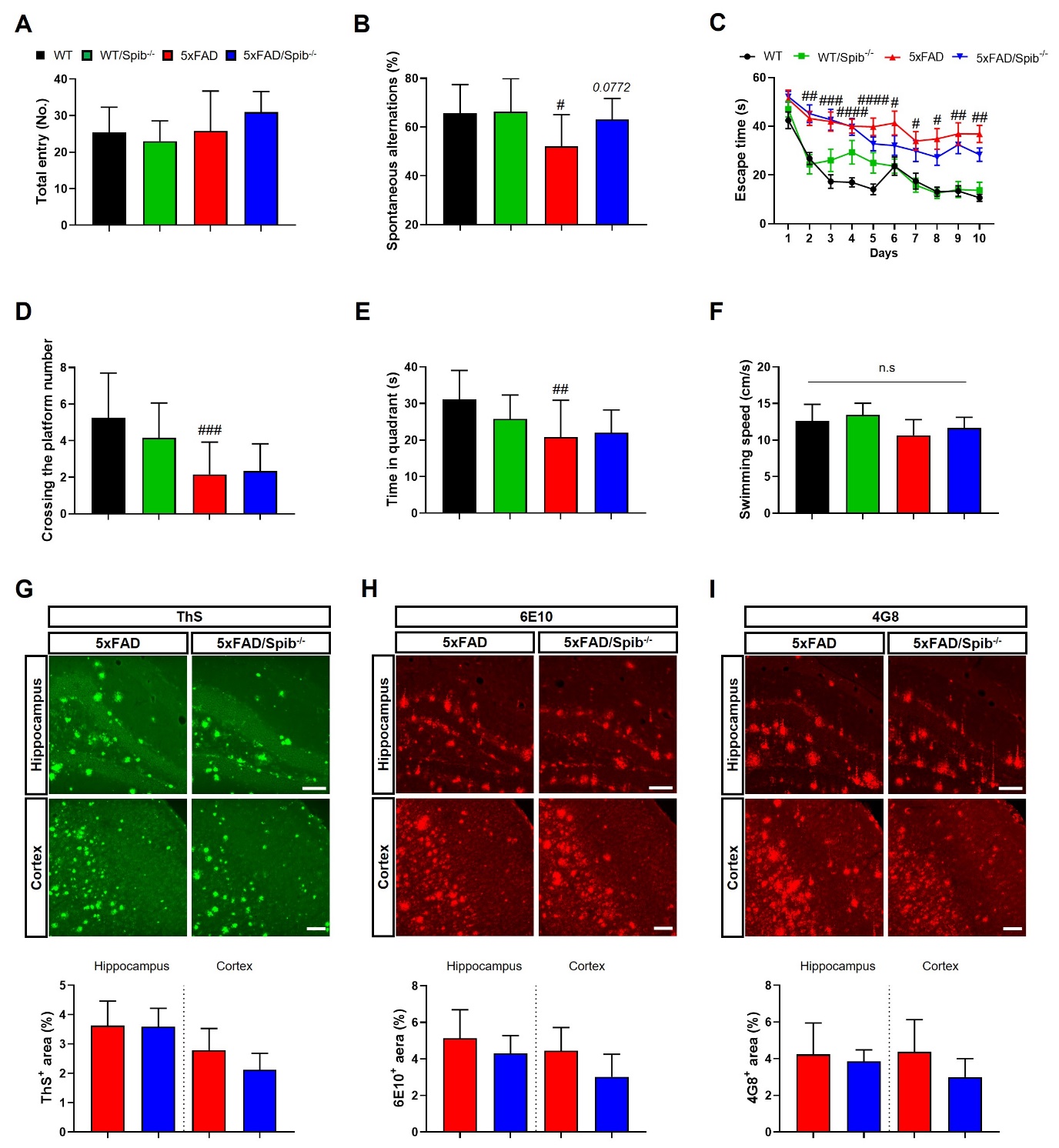


**Figure S6.** Experimental animals at 9 months did not affect the behavioral function and Aβ accumulation. (A and B) Total entry (A) and spontaneous alternations (%) (B) in Y-maze test of WT, WT/*Spib*^-/-^, 5xFAD, and 5xFAD/*Spib*^-/-^ mice (WT, *n* = 17; WT/*Spib*^-/-^, *n* = 17; 5xFAD, *n* = 18; 5xFAD/*Spib*^-/-^ mice, *n* = 16). (D–F) On day 11 of the probe trial, crossing the platform number (D), spending time in the target quadrant (E), and swimming speed (F) were recorded and analyzed. Statistical analysis included the two-way ANOVA and Tukey’s *post hoc* test. ^#^*P* < 0.05, ^##^*P* < 0.01, ^###^*P* < 0.001, and ^####^*P* < 0.0001 *versus* WT mice. (G–I) Representative images and quantification of ThS^+^ (G), 6E10^+^ (H), and 4G8^+^ (I) immunostaining in the hippocampus and cortex (*n* = 6 per group); scale bar, 100 μm. Bars represent the mean ± standard deviation. Bars of Fig. 1C represent the mean ± standard error of the mean. Statistical analysis included the Student’s *t* test.

**
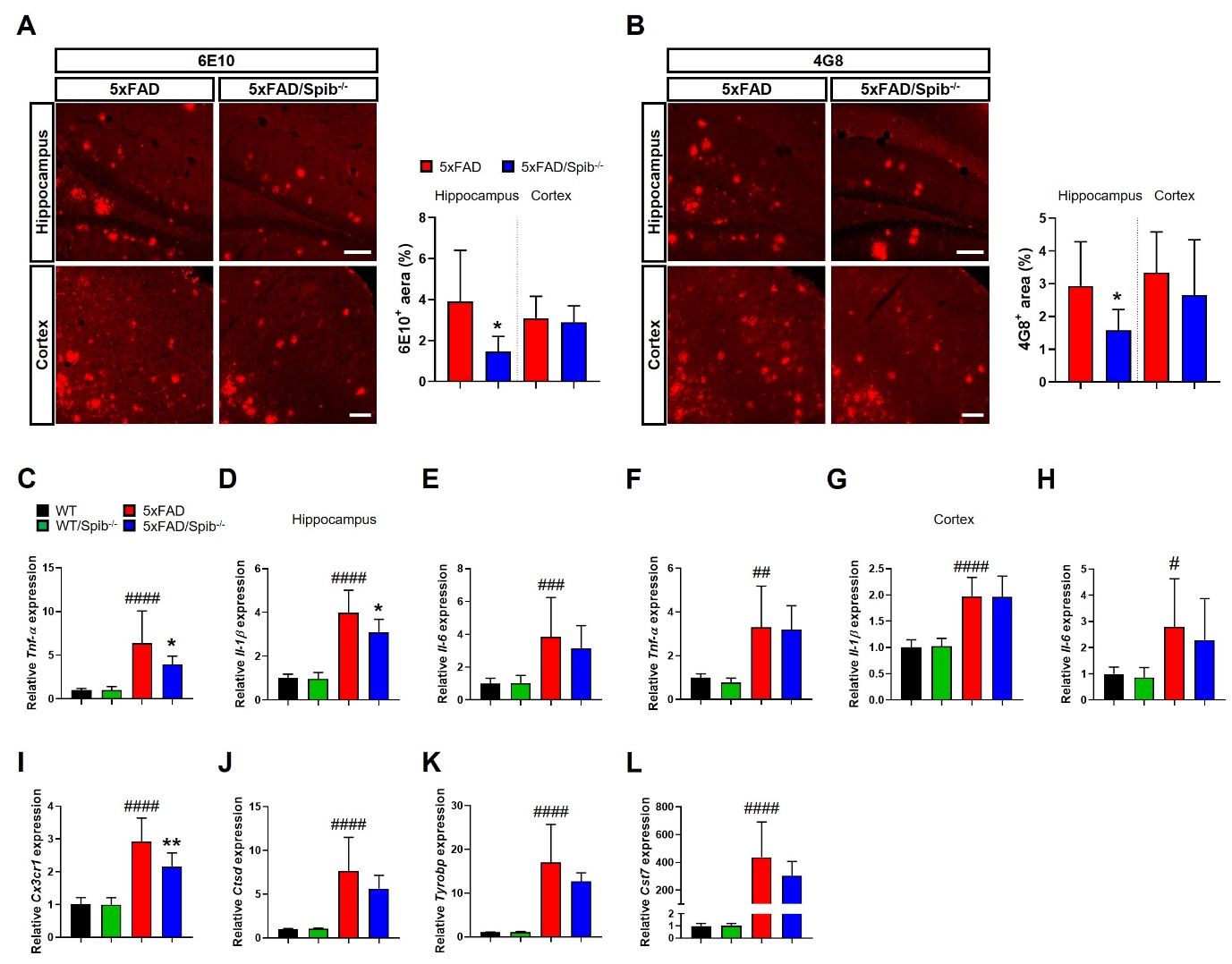
**

**Figure S7.** The effect of M cell depletion on Aβ deposition and neuroinflammation in 5xFAD/*Spib*^-/-^ mice. (A and B) Immunofluorescence images and quantification of 6E10 (A) and 4G8 (B)-positive area (%) (*n* = 6 per group); scale bar, 100 μm. Statistical analysis included the Student’s *t*-test. (C–L) The inflammatory cytokines were measured in the hippocampus (C–E and I–L) and cortex (F–H) using qRT-PCR (*n =* 7–10 per group). Bars represent as mean ± standard deviation. Statistical analysis included the two-way ANOVA and Tukey’s *post hoc* test. ^#^*P <* 0.05, ^##^*P <* 0.01, ^###^*P <* 0.001, and ^####^*P <* 0.0001 *versus* WT mice. **P* < 0.05 and ***P* < 0.01 *versus* 5xFAD mice.


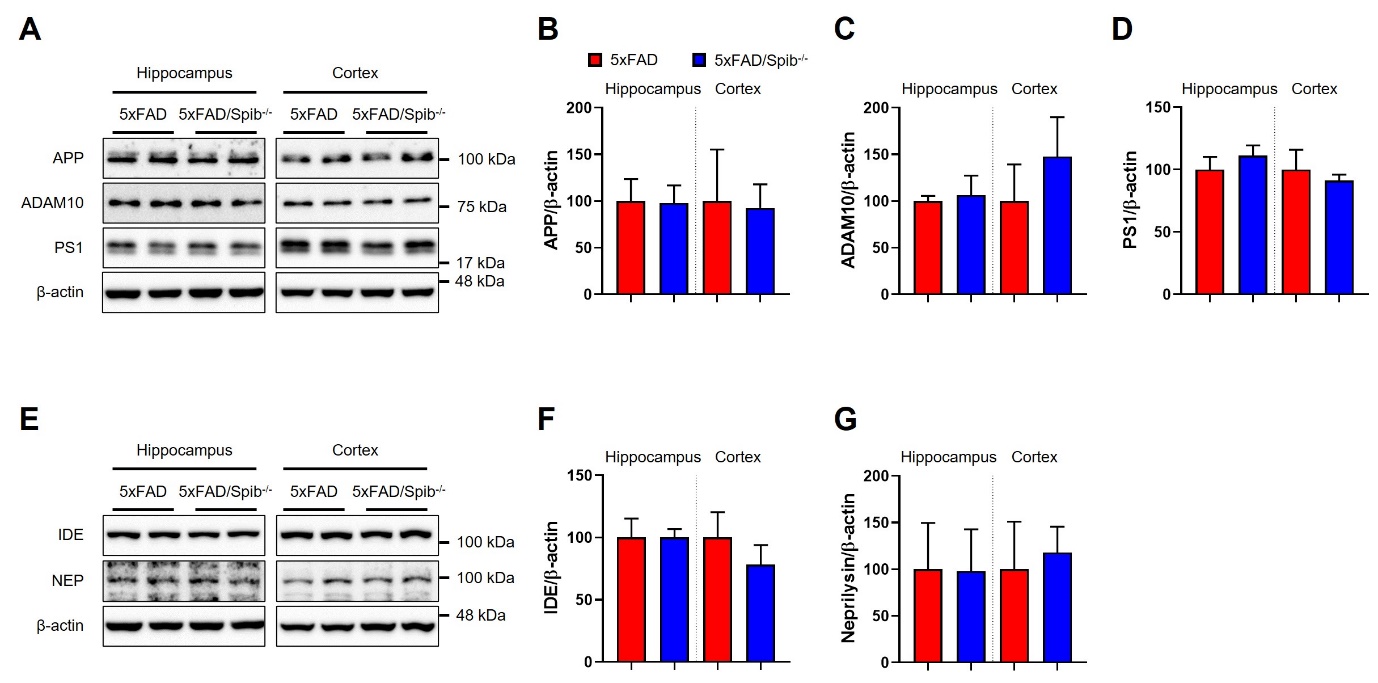


**Figure S8.** Inhibition of microfold cells did not affect the APP pathway. (A and E) Western blot analysis of Aβ process signal (A) and Aβ degrading enzymes (E) in the hippocampus and cortex of 6-month-old mice. (B–D) Quantification of APP (B), ADAM10 (C), and PS1 (D) by normalizing levels to β-actin (*n* = 3–5 per group). (F and G) Quantification of IDE (F) and NEP (G) by normalizing levels to β-actin. Bars represent the mean ± standard deviation. Statistical analysis included the Student’s *t*-test.


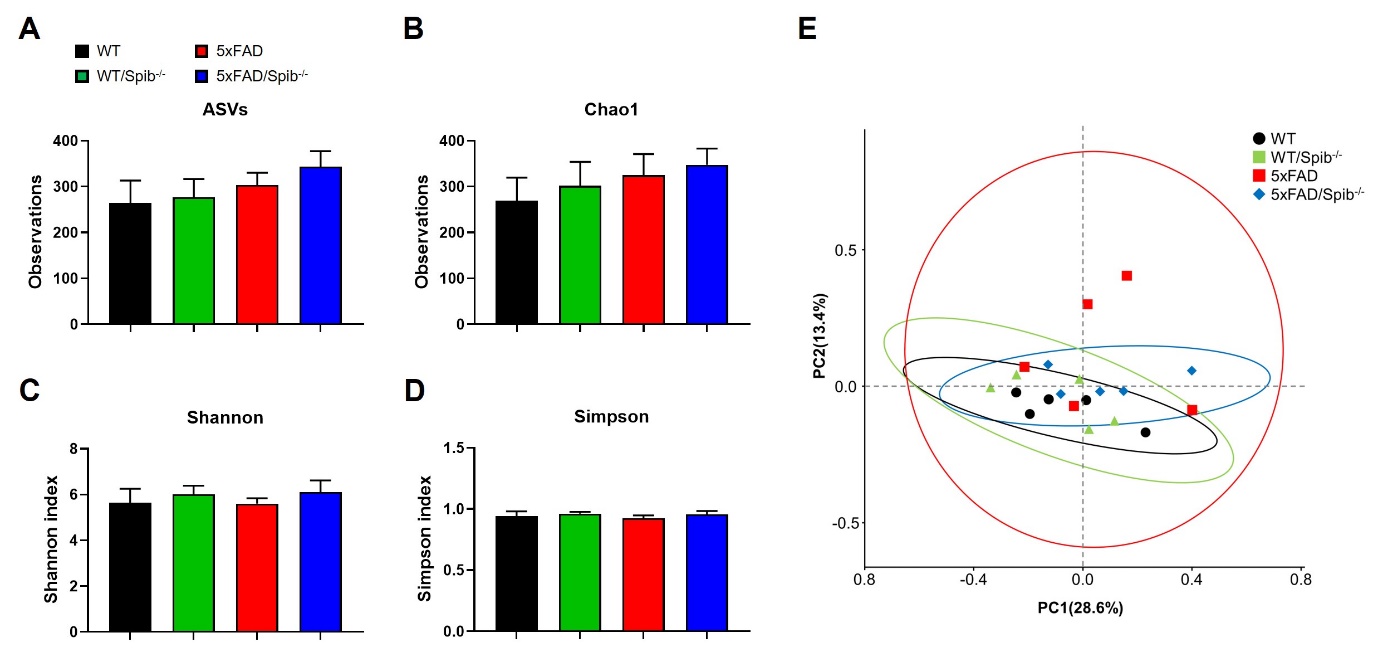


**Figure S9.** Inhibition of microfold cells did not affect the diversity of gut microbiota in 5xFAD mice. (A and B) Analysis of alpha diversity-predicted diversity of gut microbiota by ASVs (A) and Chao1 (B). (C and D) Analysis of beta diversity-predicted diversity of gut microbiota by Shannon (C) and Simpson (D). (E) Principal coordinates analysis plot of microbiota community structure (*n* = 5 per group). Bars represent the mean ± standard deviation. Statistical analysis included the two-way ANOVA and Tukey’s *post hoc* test.


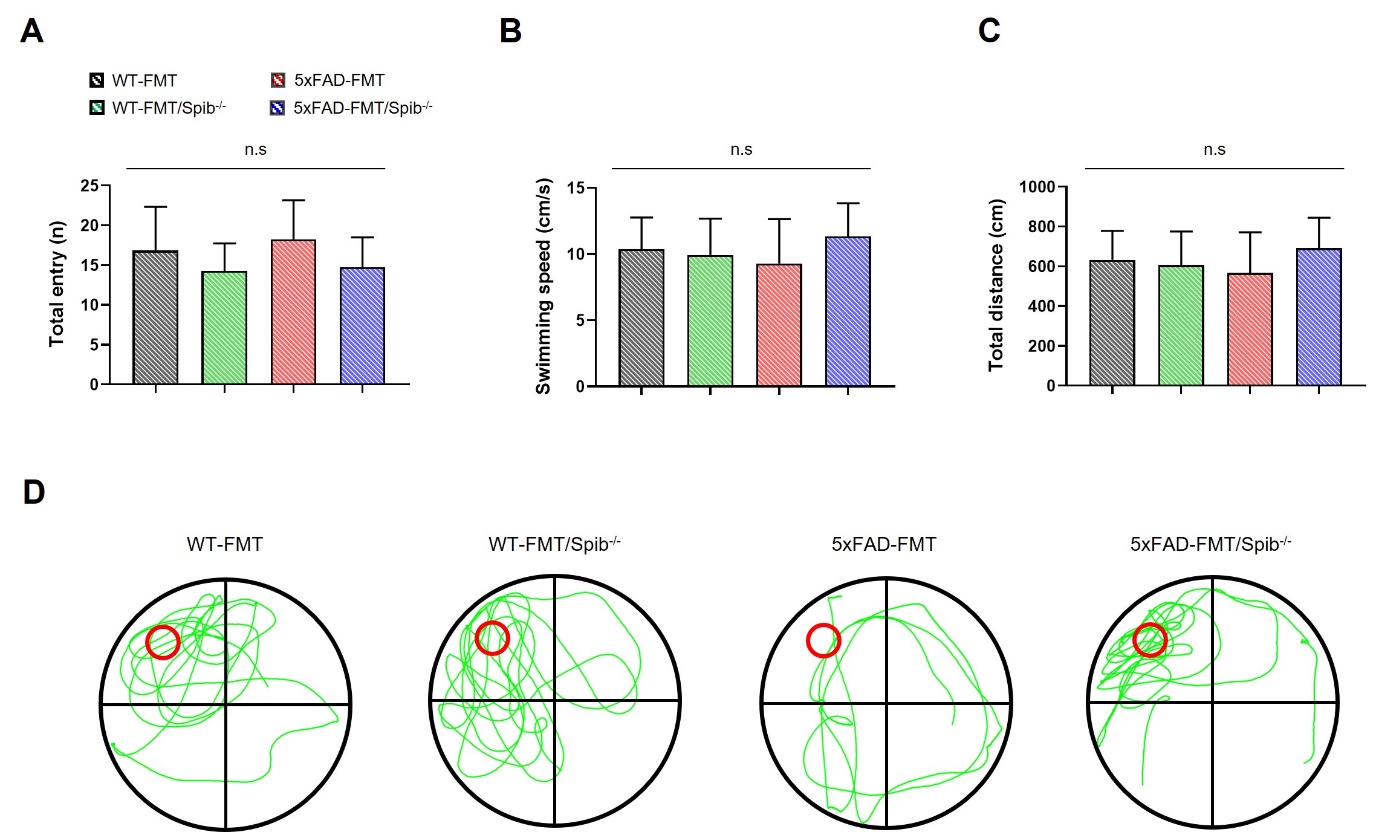


**Figure S10.** *Spib*^-/-^ mice did not reduce locomotor activity. (A) Total entry in Y-maze test of WT-FMT, WT-FMT/*Spib*^-/-^, 5xFAD-FMT, and 5xFAD-FMT/*Spib*^-/-^ mice (WT-FMT, *n* = 16; WT-FMT/*Spib*^-/-^, *n* = 15; 5xFAD-FMT, *n* = 19; and 5xFAD-FMT/*Spib*^-/-^ mice, *n* = 17). (B and C) Swimming speed (B) and total distance (C) of WT-FMT, WT-FMT/*Spib*^-/-^, 5xFAD-FMT, and 5xFAD-FMT/*Spib*^-/-^ mice on day 7 of the probe trial. (D) Representative swimming paths on day 7 of training. Bars represent the mean ± standard deviation. Statistical analysis included the two-way ANOVA and Tukey’s *post hoc* test. n.s: nonsignificant.

**Table S1. Diet information**

| **Ingredients** |  |
| --- | --- |
| Crude Protein | 20.5 % |
| Crude Fat | 3.5 % |
| Crude Fiber | 8.0 % |
| Crude Ash | 8.0 % |
| Calcium | 0.5 % |
| Phosphorus | 0.5 % |

**Table S2.** **Primer sequences**

| **Primer** | **Sequence** |
| --- | --- |
| *Gapdh* | GGC ACA GTC AAG GCT GAG AAT G |
|  | ATG GTG GTG AAG ACG ACG CCA GTA |
| *Gp2* | GAT ACT GCA CAG ACC CCT CCA |
|  | GCA GTT CCG GTC ATT GAG GTA |
| *Spib* | AGC GCA TGA CGT ATC AGA AGC |
|  | GGA ATC CTA TAC ACG GCA CAG G |
| *Marcksl1* | TTT TGC CCT CCT GTG GAT TCT |
|  | CCA CTA GGC ACA GCA CAA GAG A |
| *Annexin V* | TTT CCG TTG CAC GGA GTT GT |
|  | TTT CCT GGC GCT GAG CAT T |
| *Sgne-1* | ACG GTT AAA AAT GGC CTC AAG G |
|  | AAG GAC CCA GAT GCT GAA GAC C |
| *Ccl9* | TAC TGC CCT CTC CTT CCT CA |
|  | TTG AAA GCC CAT GTG AAA CA |
| *M-sec* | GTG CAG AAC CTC TAC CCC AAT G |
|  | TGG AGA ATG TCG ATG GCC A |
| *Rank* | AAA CCT TGG ACC AAC TGC AC |
|  | ACC ATC TTC TCC TCC CHA GT |
| *Rankl* | CGC TCT GTT CCT GTA CTT TCG AGC G |
|  | CG TGC TCC CTC CTT TCA TCA GGT T |
| *Tnf-α* | GAT TAT GGC TCA GGG TCC AA |
|  | GCT CCA GTG AAT TCG GAA AG |
| *Il-1β* | CCC AAG CAA TAC CCA AAG AA |
|  | GCT TGT GCT CTG CTT GTG AG |
| *Il-6* | CCG GAG AGG AGA CTT CAC AG |
|  | TTG CCA TTG CAC AAC TCT TT |
| Total bacteria | ACT CCT ACG GGA GGC AGC AG |
|  | ATT ACC GCG GCT GCT GG |
| *Cx3cr1* | CTT CAT CAC CGT CAT CAG CA |
|  | TGT GAA CAT GAA CTG GGG TG |
| *Ctsd* | GTC ACT CGA AAG GCC TAC TG |
|  | AAG AGA TGT CCC TGT GTC CA |
| *Tyrobp* | ACT TGG TGT TGA CTC TGC TG |
|  | CTG AAG CTC CTG ATA AGG CG |
| *Cst7* | TTT TGA GGT GCC TGT TCT CC |
|  | TGG GGT GGA AAG TAA GGA GT |
